# DeepGVS: a bimodal deep learning framework integrating coding-sequence and protein-structural representations for virulence factor prediction

**DOI:** 10.1093/bioinformatics/btag698

**Published:** 2026-09-21

**Authors:** Yan Miao, Tingting Zou, Zhenyuan Sun, Yuming Zhao, Guohua Wang

**Affiliations:** School of Computer Science and Artificial Intelligence, Northeast Forestry University, Harbin, Heilongjiang Province 150040, China; School of Computer Science and Artificial Intelligence, Northeast Forestry University, Harbin, Heilongjiang Province 150040, China; School of Computer Science and Artificial Intelligence, Northeast Forestry University, Harbin, Heilongjiang Province 150040, China; School of Computer Science and Artificial Intelligence, Northeast Forestry University, Harbin, Heilongjiang Province 150040, China; School of Computer Science and Artificial Intelligence, Northeast Forestry University, Harbin, Heilongjiang Province 150040, China; Faculty of Computing, Harbin Institute of Technology, Harbin, Heilongjiang Province 150001, China

## Abstract

**Motivation:**

Virulence factors (VFs) mediate host adhesion, invasion, immune evasion and toxin-mediated damage, making accurate VF prediction important for understanding bacterial pathogenesis and antimicrobial intervention. Existing predictors mainly use one-dimensional (1D) protein sequences, overlooking complementary coding DNA sequence (CDS)-level information and three-dimensional (3D) structural topology.

**Results:**

We propose DeepGVS, a bimodal deep learning framework integrating CDS-derived features with protein sequence and structural representations for VF prediction. DeepGVS extracts multi-scale sequence-composition features from CDS. Concurrently, it employs a parallel graph attention network (GAT) and bidirectional Mamba (Bi-Mamba) architecture to process ESMFold-predicted structures and residue representations, capturing spatial and long-range dependencies. A neural additive model (NAM) functions as a meta-learner to integrate base-classifier predictions. DeepGVS was evaluated on the unchanged accession-level independent test set of Dataset_B and achieved an accuracy of 87.50%, corresponding to absolute improvements of 6.30, 2.60 and 1.40 percentage points over the published benchmark values of DeepVF, GTAE-VF and PLMVF, respectively. The incremental benefit of bimodal integration was dataset- and metric-dependent, and additional taxonomic analyses identified taxonomy as a potential confounding factor that does not fully reproduce the performance of the complete model.

**Availability and implementation:**

Source code, datasets and pretrained models are available at https://github.com/guoguo26/DeepGVS. The archived code version used for the reported experiments is available at https://doi.org/10.5281/zenodo.22764397.

## 1 Introduction

With the continuous emergence of new infectious diseases (Becker *et al.* 2006) and the evolving pathogenic profiles of existing microbes, pathogenic infections remain a critical challenge to global public health. The global antimicrobial resistance (AMR) crisis has been exacerbated by the widespread misuse of antibiotics (Hamid and Friedberg 2019), necessitating the development of non-traditional antibacterial strategies. Virulence factors (VFs), key effector molecules that mediate pathogen colonization, immune evasion (Cross 2008), and disruption of host cellular structures (Shende *et al*. 2017), have emerged as promising targets for new drugs and vaccines. Consequently, the rapid and accurate computational identification of VFs from large-scale genomic data is important for understanding bacterial pathogenesis and supporting systematic VF annotation.

In recent years, the accumulation of high-throughput sequencing data and the establishment of specialized databases, such as VFDB (Liu *et al.* 2022), Victors (Sayers *et al.* 2019), and PATRIC (Wattam *et al.* 2017), have catalyzed the development of computational VF predictors. Early approaches based on BLAST or hidden Markov models (HMMs) relied predominantly on the detection of homologous sequences (El-Gebali *et al.* 2019, Liu *et al.* 2019). Although these methods are effective for identifying known or highly homologous VFs, their performance is limited by the coverage of reference databases and may decline when detecting remote homologs or previously uncharacterized VFs (Xie *et al.* 2021). To reduce reliance on alignment-based approaches, machine-learning methods based on manually engineered features were subsequently developed, including VirulentPred (Garg and Gupta 2008) and MP3 (Gupta *et al.* 2014). However, the performance of these methods depends on the choice and design of handcrafted features, which may limit their ability to represent complex sequence patterns.

The field of VF prediction has been significantly advanced by deep learning, owing to its capacity for automated representation learning. For instance, tools such as DeepVF (Xie *et al.* 2021) and PreVFs-RG (Zhang and Jing 2023) learn high-level representations from protein sequences. More recently, predictors based on pretrained protein language models (PLMs), such as DTVF (Sun *et al.* 2024), have utilized ProtT5 (Elnaggar *et al.* 2022) to generate context-aware protein embeddings. Nevertheless, these approaches predominantly operate at the amino-acid sequence level and do not explicitly represent the three-dimensional spatial organization of proteins. Structure-aware methods have therefore emerged. GTAE-VF (Li *et al.* 2024), e.g. combines ESMFold-predicted protein structures (Lin *et al.* 2023) with Graph Neural Networks (GNNs) (Scarselli *et al.* 2009) to extract structure-aware representations. Similarly, PLMVF (Li *et al.* 2025) integrates ESM-2 (Lin *et al.* 2023) sequence representations with structural information derived from ESMFold-predicted structures. These studies indicate that structural information can provide complementary information to protein sequence representations for VF prediction.

Despite these advances, two aspects remain insufficiently explored. First, protein function reflects information at different representational scales, including local residue organization in three-dimensional space and long-range dependencies along the amino-acid sequence. Although recent VF predictors have incorporated both sequence- and structure-derived information, the complementary contributions of residue-level spatial topology and long-range sequential context within a unified protein representation remain incompletely characterized. Second, most current VF predictors operate primarily at the amino-acid level and do not explicitly use nucleotide-level information retained in the corresponding CDSs. Horizontal gene transfer can introduce virulence-associated genomic regions with distinct nucleotide-composition characteristics, including GC content and codon-usage patterns (Garcia-Vallvé *et al.* 2003, Gal-Mor and Finlay 2006). Because some nucleotide-level information is not explicitly retained after translation, CDS-derived characteristics may provide predictive information complementary to protein-derived representations. However, nucleotide composition, codon usage, and related CDS characteristics can also vary systematically across species and phylogenetic lineages and are therefore not necessarily specific to virulence. The contribution of CDS-derived information should consequently be interpreted in the context of potential taxonomic structure.

To address these issues, we propose DeepGVS, a bimodal deep learning framework that integrates CDS-derived features with protein sequence–structure representations. DeepGVS extracts multi-scale sequence features from the CDS modality, including nucleotide-composition, short-range sequence-pattern, regional-heterogeneity, and low-complexity characteristics. In the protein modality, ESMFold-derived residue graphs and ESM-2 residue representations are processed using a parallel architecture combining a Graph Attention Network (GAT) (Veličković *et al.* 2018) and Bidirectional Mamba (Bi-Mamba) (Gu and Dao 2024, Schiff *et al.* 2024). The GAT models local spatial relationships between residues, whereas Bi-Mamba captures long-range sequential dependencies, and the resulting representation is complemented by a global ProtT5 embedding. CDS- and protein-derived representations are then supplied to multiple base classifiers, whose predictions are integrated using a Neural Additive Model (NAM) meta-learner. DeepGVS was evaluated on two protein–CDS paired benchmark datasets, and its predictive performance was compared with representative sequence-, language-model-, and structure-based VF predictors, including DeepVF, GTAE-VF, and PLMVF. We further conducted modality- and architecture-specific ablation analyses, examined the potential influence of taxonomic structure, and assessed the practical utility and interpretability of the framework through proteome-wide screening and SHAP-based feature attribution analysis.

## 2 Methods

The workflow of DeepGVS is illustrated in Fig. 1a, which comprises four core modules: Dataset construction and curation (Fig. 1b), CDS feature extraction (Fig. 1c), protein sequence–structure feature extraction (Fig. 1d), and NAM-based ensemble prediction (Fig. 1e).

**Figure 1:**
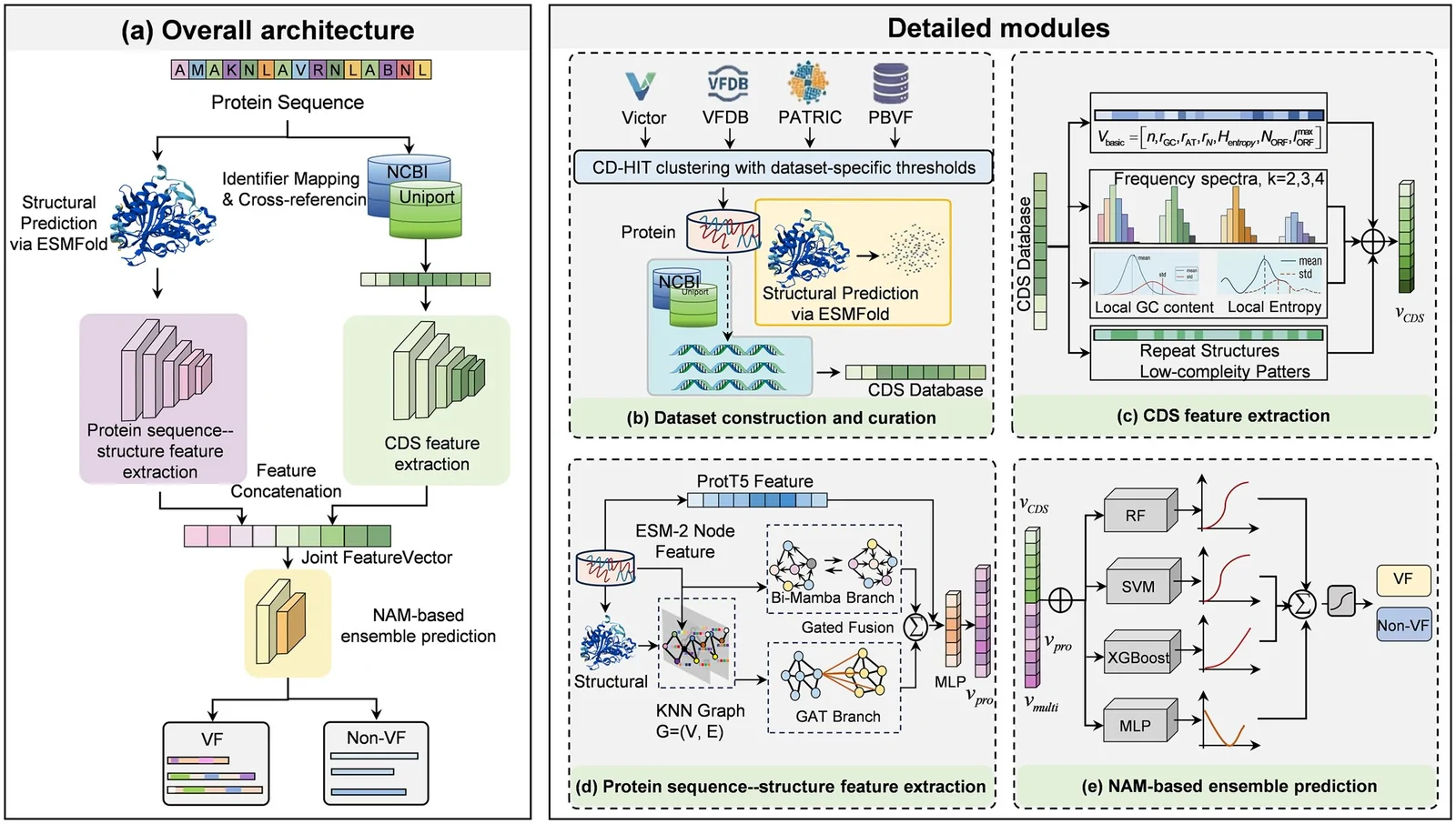
Overview of DeepGVS. (a) Overall framework for VF prediction. (b) Dataset construction and curation. (c) CDS feature extraction. (d) Protein sequence–structure feature extraction. (e) NAM-based ensemble prediction.

### 2.1 Dataset construction and curation

To train and evaluate DeepGVS, two bimodal datasets (Dataset_A and Dataset_B) were used. Each sample comprises a protein sequence and its corresponding CDS. Sequences lacking a clear one-to-one protein-to-CDS mapping were excluded.

For Dataset_A, 4263 experimentally verified virulence factors (VFs; positive samples) were retrieved from the VFDB Core Set A (accessed 18 April 2025) (Liu *et al.* 2022). Non-VFs (negative samples) were generated using a previously described whole-proteome sampling strategy (Rentzsch *et al.* 2020). CD-HIT (Li and Godzik 2006) was applied with a sequence-identity cutoff of 0.5. VF CDSs were retrieved from the VFDB nucleotide database, whereas non-VF CDSs were retrieved from NCBI, UniProt, or ENA using protein accession information. Following redundancy reduction, CDS mapping, and class balancing, Dataset_A contained 7168 paired protein–CDS samples (3584 VFs and 3584 non-VFs), partitioned into training (4999), validation (800), and independent test (1369) sets.

Dataset_B retained the original DeepVF benchmark partitioning (Xie *et al.* 2021). In the original benchmark, CD-HIT filtering at a sequence-identity cutoff of 0.3 was performed before establishment of the development pool and accession-level independent test set. We did not rerun CD-HIT or reconstruct the test set. The unchanged independent test set contained 576 VFs and 576 non-VFs. From the original development pool of 3000 VFs and 4334 non-VFs, we retained all 3000 VFs and selected 3000 non-VFs to form a balanced development set, which was divided into training (5400) and validation (600) sets. CDS retrieval was performed after the predefined protein-level partitions were fixed and did not alter test membership.

The training, validation, and independent test sets were used for model fitting, parameter selection, and final evaluation, respectively; detailed class compositions are provided in Table S1. Because CDS-derived features may also reflect taxonomic background, we additionally evaluated taxonomic composition, taxonomy-only logistic-regression baselines, and within-taxon sensitivity analyses. Taxonomic annotations were used only for these post hoc analyses and were not inputs to DeepGVS (Supplementary Methods S4; Supplementary Tables S8–S12; Supplementary Data S1–S4).

### 2.2 CDS feature extraction

For each CDS, DeepGVS extracted four groups of multi-scale nucleotide features: global statistical features, *k*-mer frequencies for k∈{2,3,4}, sliding-window heterogeneity features, and repeat or low-complexity features, capturing nucleotide composition, entropy, ORF-related attributes, and regional heterogeneity. Local heterogeneity features were calculated using a 128-bp window with a 128-bp step size. The unified CDS representation was defined as


(1) $$v_{\text{CDS}}=v_{\text{basic}}⊕v_{\text{kmer}}⊕v_{\text{window}}⊕v_{\text{repeat}},$$

where ⊕ denotes vector concatenation. Detailed feature definitions and dimensions are provided in Table S2.

### 2.3 Protein sequence–structure feature extraction

For each protein, ESMFold-predicted $C_{\alpha }$ coordinates were used to construct a residue-level spatial graph by connecting each residue to its 30 nearest spatial neighbors according to Euclidean distance. The graph was symmetrized, self-loops were removed, and binary edge weights were used. Nodes were initialized with 256-dimensional ESM-2 embeddings. A parallel GAT and Bi-Mamba encoder captured local spatial interactions and long-range sequence dependencies, respectively, and the two outputs were integrated by gated fusion (Supplementary Methods S2).

The fused representation was pooled and concatenated with the ProtT5-derived global embedding $t_{\text{ProtT}5}$ to obtain the final protein representation:


(2) $$v_{\text{pro}}=\text{MLP}\left(\text{Pool}(h_{\text{fuse}})\|t_{\text{ProtT}5}\right).$$

Protein sequences longer than 1000 amino acids were truncated to the first 1000 residues; no sliding-window or segment-merging procedure was used for protein sequences. All proteins in both datasets were successfully processed by ESMFold. Additional implementation, optimization, software/hardware, and resource-usage details are provided in Supplementary Methods S3 and Table S13.

### 2.4 NAM-based ensemble prediction

The bimodal representation $v_{\text{multi}}=[v_{\text{CDS}}\|v_{\text{pro}}]$ was supplied to four base classifiers: Random Forest, SVM, XGBoost, and MLP. Their predicted VF probabilities $p_{k}$ were integrated by a Neural Additive Model (NAM):


(3) $$P(\text{VF})=\sigma \left(\beta +\sum_{k=1}^{4}f_{k}(p_{k})\right),$$

where $f_{k}(\cdot )$ is the learnable shape function for the *k*-th classifier, β is the global bias, and P(VF) is the final virulence probability.

The NAM meta-learner was trained using five-fold stratified out-of-fold probability predictions from the base classifiers. The base classifiers were then refitted on the complete development set before independent-test prediction. The global random seed was 42; detailed base-classifier and NAM settings are provided in Supplementary Methods S3 and Table S13.

A fixed probability threshold of 0.5 was used for ACC, REC, SPE, PRE, F1-score, and MCC, whereas AUROC and AUPRC were calculated from continuous prediction probabilities. The same 0.5 threshold was used for proteome-wide screening; metric definitions are provided in Supplementary Methods S1.

## 3 Results

### 3.1 Predictive performance and comparison with published methods

On the independent test set of Dataset_A, DeepGVS demonstrated strong predictive capability, achieving an ACC of 0.9262, AUROC of 0.9795, and AUPRC of 0.9766 (detailed metrics in Table S3).

On the unchanged Dataset_B independent test set, DeepGVS achieved an ACC of 87.50% (Table 1), outperforming the published benchmark values of DeepVF, GTAE-VF, and PLMVF by 6.30, 2.60, and 1.40 percentage points, respectively. While PLMVF reported a higher REC, DeepGVS achieved a superior balance between specificity (90.10%) and overall F1-score (0.8717). Note that baseline values are cited from original publications rather than a unified reimplementation, serving as benchmark-level evidence rather than a controlled head-to-head evaluation.

**Table 1:** Performance comparison of DeepGVS and published VF prediction methods on the independent test set of Dataset_B.

Method	ACC	REC	SPE	F1	AUROC	MCC
	(%)	(%)	(%)			
VirulentPred (Garg and Gupta, 2008)	60.70	64.10	57.30	0.6200	–	0.2140
MP3 (Gupta *et al.* 2014)	66.00	53.60	78.30	0.6120	–	0.3300
BLAST (Xie *et al.* 2021)	75.00	68.20	81.80	0.7320	–	0.5050
PBVF (Rentzsch *et al.* 2020)	79.40	77.40	81.40	0.7900	0.8790	0.5890
DeepVF (Xie *et al.* 2021)	81.20	79.00	83.30	0.8070	0.8960	0.6240
VF-Pred (Singh *et al.* 2024)	83.51	87.00	76.00	0.8257	0.8160	–
DTVF (Sun *et al.* 2024)	84.55	80.21	88.89	0.8385	0.9208	–
GTAE-VF (Li *et al.* 2024)	84.90	81.40	87.90	0.8340	–	–
PLMVF (Li *et al.* 2025)	86.10	**87.30**	87.70	0.8590	0.9198	–
DeepGVS (protein-only)	86.81	84.20	89.41	0.8645	0.9269	0.7371
**DeepGVS**	**87.50**	84.90	**90.10**	**0.8717**	**0.9275**	**0.7510**

Baseline values were taken from the respective original publications rather than obtained through a unified reimplementation; differences in training data, preprocessing, and implementation may therefore remain. “–” indicates an unavailable metric. Bold values indicate the best performance for each evaluation metric among the compared methods.

### 3.2 Taxonomic composition and potential confounding

VF and non-VF samples differed in taxonomic composition (Table S8; Supplementary Data S1–S3). A taxonomy-only logistic-regression model using combined family, genus, and species information achieved AUROC = 0.933 on Dataset_A and 0.868 on Dataset_B, compared with 0.9795 and 0.9275 for DeepGVS, respectively (Table S9). These results indicate that taxonomic composition contributes to the observed discrimination but does not fully account for DeepGVS performance. Within-taxon analyses also retained discrimination in eligible taxa (Supplementary Tables S10–S11; Supplementary Data S4). However, the benchmark splits were not taxonomically disjoint, and finer strain- or lineage-related effects may remain.

### 3.3 Model ablation and interpretability analyses

#### 3.3.1 Ablation study of bimodal fusion

We compared CDS-only, protein-only, and bimodal models (Fig. S1; Tables S3–S4). The incremental benefit of bimodal integration varied by dataset. On Dataset_A, fusion primarily enhanced SPE (from 0.8932 to 0.9473) while CDS-only retained a slightly higher REC. On Dataset_B, the protein-only model already performed strongly (AUROC = 0.9269); thus, bimodal fusion provided modest but consistent improvements across threshold-dependent metrics and AUPRC. (Feature-space visualizations are shown in Supplementary Figs. S2–S3).

The five-fold cross-validation ACC on Dataset_B (∼0.9823) was higher than the independent-test ACC (0.8750). Because CV folds were random stratified partitions without re-running CD-HIT internally, we regard the independent test as the more conservative and reliable estimate of generalization.

#### 3.3.2 Ablation study on the protein modality architecture

Table 2 illustrates the contribution of the protein encoder components. On both datasets, the parallel integration of GAT and Bi-Mamba improved overall performance compared to either standalone module. The addition of ProtT5 global embeddings further maximized ACC and MCC, confirming the complementary value of local spatial topology, long-range sequence dependencies, and global protein-language context. (Note: Table 1 results used Stacking-NAM, whereas Table 2 reflects direct classification from the protein encoder; see Supplementary Methods S3).

**Table 2:** Ablation study on the core architecture of the protein modality.

Dataset	Model	ACC	F1	REC	SPE	MCC
Dataset_A	GAT	0.8773	0.8735	0.8467	0.9079	0.7560
	Bi-Mamba	0.8795	0.8788	0.8730	0.8860	0.7590
	GAT + Bi-Mamba	0.8955	0.8932	0.8730	**0.9181**	0.7919
	GAT + Bi-Mamba + ProtT5	**0.8970**	**0.8975**	**0.9007**	0.8933	**0.7940**
Dataset_B	GAT	0.8264	0.8235	0.8385	0.8142	0.6530
	Bi-Mamba	0.8446	0.8408	0.8211	0.8680	0.6899
	GAT + Bi-Mamba	0.8446	0.8474	**0.8628**	0.8263	0.6896
	GAT + Bi-Mamba + ProtT5	**0.8585**	**0.8511**	0.8090	**0.9080**	**0.7206**

Bold values indicate the best result for each metric within each dataset.

#### 3.3.3 Evaluation of base classifiers and Stacking-NAM

The decision-level ensemble was evaluated against the four base classifiers under CDS-only, protein-only, and fused feature settings. Across these settings, Stacking-NAM provided a competitive overall balance relative to the individual classifiers. Complete comparisons and the distinction between Stacking-NAM and direct protein-encoder classification are provided in Supplementary Tables S5–S6, Fig. S5, and Supplementary Methods S3.

#### 3.3.4 SHAP-based feature contribution analysis

To interpret the fused representation, we first applied SHAP to the four base-learner probabilities entering the NAM meta-learner and selected the base learner with the largest mean absolute NAM-level contribution for feature-level analysis (Supplementary Methods S5). For the Dataset_A fused model, this procedure selected SVM-RBF (Fig. S6). Its feature-level SHAP profile included interpretable CDS-derived descriptors, such as nucleotide *k*-mers, together with learned Graph-Mamba (GM) latent dimensions (Fig. 2). Across all fused input features, the mean absolute SHAP values yielded modality shares of 24.8% for CDS-derived features and 75.2% for GM features. The same selection procedure identified Random Forest for the Dataset_B fused model.

**Figure 2:**
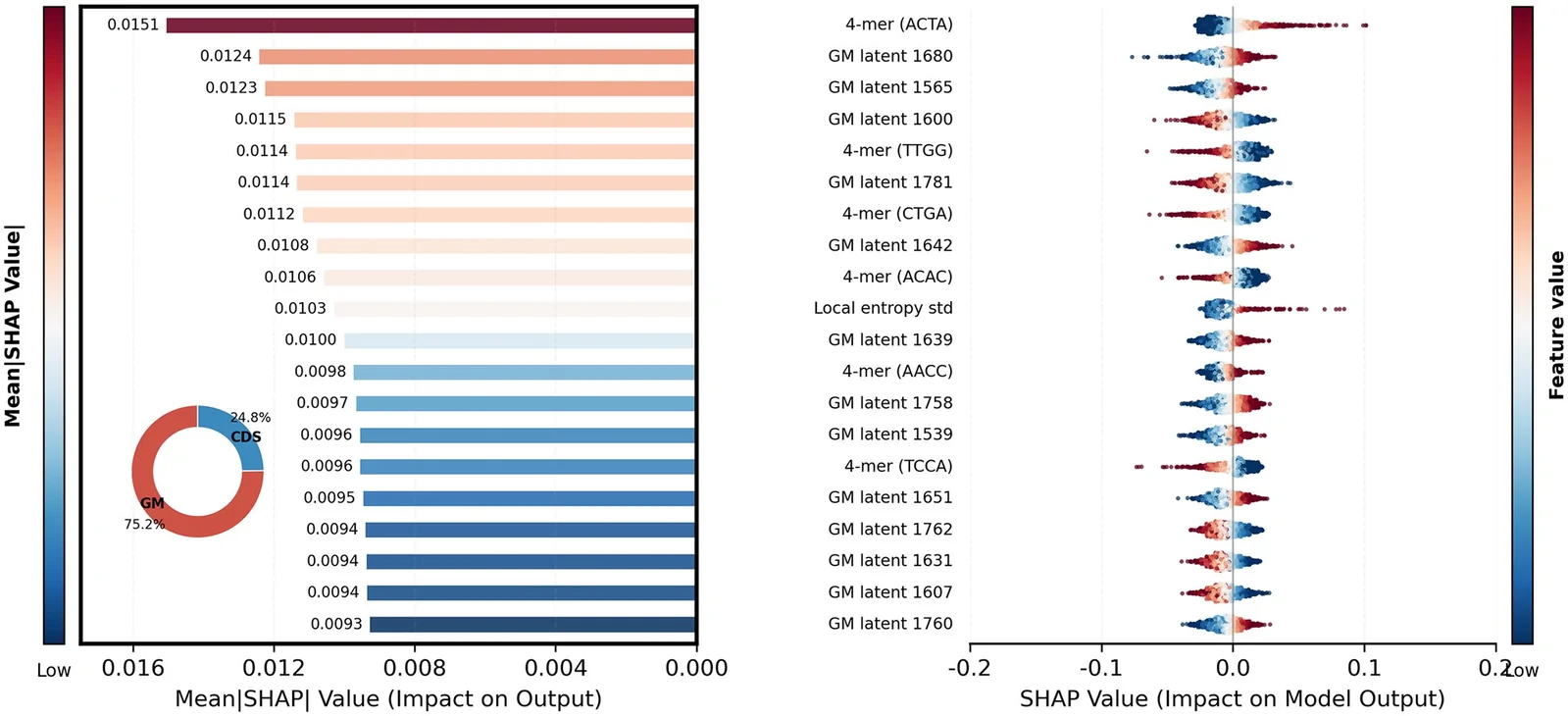
Feature-level SHAP analysis of the Dataset_A fused representation using the SVM-RBF learner selected by NAM-level attribution. The modality percentages were calculated across all fused input features by summing feature-wise mean absolute SHAP values within the CDS and GM feature groups and normalizing the two modality totals to 100%; the resulting shares were 24.8% CDS and 75.2% GM.

Additional learner-sensitivity and separately trained unimodal analyses are provided in the Supplementary Information. The CDS-only and protein-only SHAP analyses characterize independently trained unimodal predictors and are not decompositions of the fused DeepGVS prediction. GM latent dimensions are treated as learned representation coordinates and are not assigned directly to individual residues, structural regions, or molecular mechanisms.

### 3.4 Proteome-wide screening and candidate prioritization

We applied DeepGVS to the *Escherichia coli* O157: H7 proteome (5056 sequences) after excluding proteins with >30% identity to the training sets. A dual-model consensus strategy retained proteins with predicted virulence probabilities ≥0.50 from both the Dataset_A- and Dataset_B-trained models (Fig. 3a), yielding eight high-confidence computational candidates.

**Figure 3:**
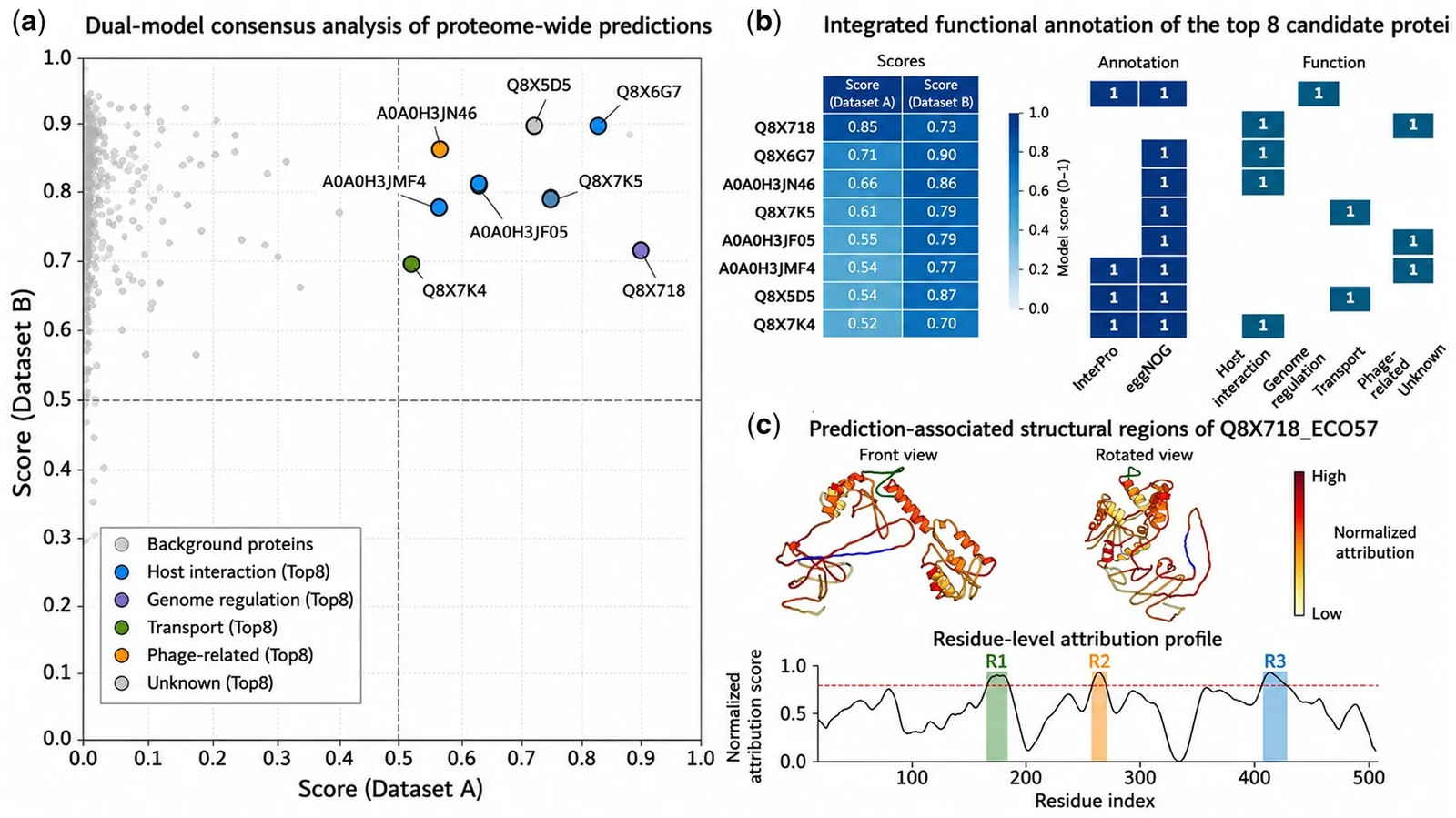
Proteome-wide screening in *E. coli* O157: H7. (a) Dual-model consensus predictions with 0.50 thresholds. (b) Annotation and functional categories of the top candidates. (c) Structural mapping of three high-attribution regions in Q8X718_ECO57: R1 (171–191), R2 (261–273), and R3 (407–430).

InterPro, UniProt, or eggNOG annotations were available for seven candidates (Fig. 3b; Table S7), including annotations associated with recombination, phage-related activity, transport, or host interaction.

For the top-ranked candidate Q8X718_ECO57, sliding-window sequence masking was used to examine local attribution patterns (Fig. 3c). Importance scores were smoothed with a seven-residue moving average, min–max normalized, and thresholded at the top 12% of the sequence-wide distribution; continuous regions shorter than four residues were excluded. This identified three high-attribution regions, R1 (171–191), R2 (261–273), and R3 (407–430), which were mapped onto the predicted structure as candidate regions for experimental follow-up rather than validated mechanistic determinants.

## 4 Discussion

DeepGVS demonstrates that integrating CDS-derived sequence composition with protein language-model and structural representations significantly advances VF prediction. By utilizing a parallel GAT and Bi-Mamba architecture, the model effectively captures both local spatial topology and long-range sequential dependencies.

Our evaluations indicate a complementary, though dataset-dependent, contribution of bimodal integration. On Dataset_B, protein representations alone yielded robust performance, suggesting that structural and language-model embeddings capture the majority of virulence-related variance in well-characterized datasets. However, bimodal fusion consistently improved specificity. This highlights the value of CDS-level features, which likely capture nucleotide composition and codon-usage patterns frequently associated with horizontally transferred virulence elements—signatures not explicitly preserved post-translation.

Furthermore, model ablation and SHAP analyses confirm the interpretability and synergistic effects of our architecture. The combination of GAT and Bi-Mamba outperformed standalone modules, while SHAP profiles (using the NAM-selected SVM-RBF base learner) successfully identified explicit CDS descriptors and learned Graph-Mamba latent dimensions as key predictive drivers. Importantly, these SHAP values characterize predictive attribution rather than direct biological mechanisms; specific structural regions prioritized by the model require further experimental validation.

Taxonomic composition is another critical consideration. Taxonomy-only models achieved high AUROC scores (0.933 on Dataset_A; 0.868 on Dataset_B), indicating substantial taxonomy-driven VF/non-VF discrimination. While broad taxonomic identity did not fully replicate DeepGVS performance—and discrimination persisted in within-taxon analyses—residual lineage-related confounding may still exist, as CDS and protein features inherently encode phylogenetic signatures.

Regarding generalization, the performance gap between internal cross-validation (ACC ∼0.9823) and the independent test (ACC 0.8750) on Dataset_B stems from the cross-validation folds being random stratified partitions rather than sequence-cluster-disjoint sets. We therefore report the independent test as the more conservative and reliable metric of model generalization.

The practical utility of DeepGVS was illustrated via proteome-wide screening of *E. coli* O157: H7. Seven of eight high-confidence candidates possessed annotations linked to recombination, phage activity, transport, or host interactions. For candidate Q8X718_ECO57, sliding-window mapping identified structural regions (e.g. residues 171–191) that serve as computationally prioritized targets for experimental follow-up.

Several limitations remain. Currently, DeepGVS is designed for binary VF prediction and does not classify functional subtypes (e.g. toxins vs. adhesins). It also processes individual sequences without modeling broader genomic contexts like pathogenicity islands or operons. Future extensions should incorporate genomic-context modeling and pathogen-specific niche adaptations, while continually benefiting from the growing completeness of high-quality VF annotations and advanced protein structure predictions.

## 5 Conclusion

In this study, we developed DeepGVS, a bimodal framework integrating CDS-derived features with protein sequence–structure representations for virulence factor (VF) prediction. The model was evaluated on two benchmark datasets, using sequence-identity cutoffs of 0.5 for Dataset A and 0.3 for the original DeepVF-based Dataset B benchmark. The model achieved competitive performance on both independent test sets, with a specificity of 90.10% on Dataset_B. Bimodal integration provided modest and dataset-dependent improvements, suggesting complementary predictive contributions from CDS- and protein-derived representations. Taxonomic analyses indicated that taxonomic composition contributed to, but did not fully explain, the observed predictive performance. Overall, DeepGVS provides a practical approach for integrating CDS- and protein-level information for VF prediction and prioritization, with future extensions to functional subtyping, genomic-context modeling, and pathogen-specific analysis.

## Supplementary Material

btag698_Supplementary_Data

## Data Availability

The data and source code underlying this article are available at https://github.com/guoguo26/DeepGVS. The archived code version used for the reported experiments is available at https://doi.org/10.5281/zenodo.22764397.
