# Supplementary material for: DeepGVS: a bimodal deep learning framework integrating coding-sequence and protein-structural representations for virulence factor prediction
Source: Bioinformatics. 2026 Sep 21;42(10):btag698. doi: 10.1093/bioinformatics/btag698 (PMC13662690; doi:10.1093/bioinformatics/btag698)
Supplement: Supplementary file 1 [file btag698_supplementary_data.zip › Supplementary_Data/DeepGVS__Supplementary_Data.pdf]

Supplementary Information

DeepGVS: a bimodal deep learning framework integrating coding-sequence and protein-structural representations for virulence factor prediction

Contents

Supplementary Methods

Item	Description	Page
Methods S1	Evaluation metrics and decision threshold	3
Methods S2	GAT, Bi-Mamba encoding, and gated fusion	3
Methods S3	Implementation, reproducibility, and Stacking-NAM details	5
Methods S4	Taxonomic composition and potential confounding analysis	6
Methods S5	SHAP calculation, feature mapping, and modality aggregation	7

Supplementary Tables

Item	Description	Page
Table S1	Dataset class composition and data splits	9
Table S2	Definitions and dimensions of CDS-derived features	10
Table S3	Five-fold cross-validation of unimodal and bimodal models	11
Table S4	Independent test performance of unimodal and bimodal models	11
Table S5	Five-fold cross-validation of base classifiers	12
Table S6	Independent test comparison of base classifiers and Stacking-NAM	13
Table S7	eggNOG annotations of candidate proteins	14
Table S8	Taxonomic annotation coverage	15
Table S9	Taxonomy-only baseline performance	15
Table S10	Within-taxon independent-test sensitivity summary	15
Table S11	Dataset A within-species sensitivity results	16
Table S12	Paired bootstrap comparison of taxonomy-only and DeepGVS predictions	16
Table S13	Model hyperparameters, software, hardware, runtime, and memory	17
Table S14	Global SHAP modality aggregation for primary and sensitivity fused-model learners	17

Supplementary Figures

Item	Description	Page
Figure S1	Independent test performance of ablation models	18
Figure S2	Feature-space visualization on Dataset A	19
Figure S3	Feature-space visualization on Dataset B	20
Figure S4	Protein-modality architecture comparison	20
Figure S5	Cross-validation MCC distributions of base classifiers and Stacking-NAM	21
Figure S6	NAM meta-feature SHAP for the Dataset A fused stacking model	21
Figure S7	Primary SVM-RBF fused-feature SHAP analysis on Dataset A	22
Figure S8	Fused MLP SHAP sensitivity analysis on Dataset A	22
Figure S9	NAM meta-feature SHAP for the Dataset A CDS-only stacking model	23
Figure S10	Primary XGBoost SHAP analysis of the separately trained Dataset A CDS-only model	23
Figure S11	NAM meta-feature SHAP for the Dataset A protein-only stacking model	24
Figure S12	Primary Random Forest SHAP analysis of the separately trained Dataset A protein-only model	24
Figure S13	NAM meta-feature SHAP for the Dataset B fused stacking model	25
Figure S14	Primary Random Forest fused-feature SHAP analysis on Dataset B	25
Figure S15	CDS-restricted SHAP ranking for the selected Dataset B Random Forest fused model	26
Figure S16	Fused MLP SHAP sensitivity analysis on Dataset B	26
Figure S17	NAM meta-feature SHAP for the Dataset B CDS-only stacking model	27
Figure S18	Primary XGBoost SHAP analysis of the separately trained Dataset B CDS-only model	27
Figure S19	NAM meta-feature SHAP for the Dataset B protein-only stacking model	28
Figure S20	Primary Random Forest SHAP analysis of the separately trained Dataset B protein-only model	28

Supplementary Data **29**

Supplementary Methods

Supplementary Methods S1. Evaluation metrics and decision threshold

The predictive performance of DeepGVS was evaluated using accuracy (ACC), recall (REC), specificity (SPE), precision (PRE), F1-score, Matthews correlation coefficient (MCC), area under the receiver operating characteristic curve (AUROC), and area under the precision–recall curve (AUPRC). For binary classification, true positives, true negatives, false positives, and false negatives are denoted as TP, TN, FP, and FN, respectively.

Accuracy was calculated as:

$$\mathrm{ACC} = \frac{TP + TN}{TP + TN + FP + FN}. \tag{S1}$$

Recall was calculated as:

$$\mathrm{REC} = \frac{TP}{TP + FN}. \tag{S2}$$

Specificity was calculated as:

$$\mathrm{SPE} = \frac{TN}{TN + FP}. \tag{S3}$$

Precision was calculated as:

$$\mathrm{PRE} = \frac{TP}{TP + FP}. \tag{S4}$$

The F1-score was calculated as:

$$\mathrm{F1} = \frac{2 \times \mathrm{PRE} \times \mathrm{REC}}{\mathrm{PRE} + \mathrm{REC}}. \tag{S5}$$

The Matthews correlation coefficient was calculated as:

$$\mathrm{MCC} = \frac{TP \times TN - FP \times FN}{\sqrt{(TP + FP)(TP + FN)(TN + FP)(TN + FN)}}. \tag{S6}$$

The AUROC was calculated as the area under the receiver operating characteristic curve, whereas the AUPRC was calculated as the area under the precision–recall curve.

A fixed probability threshold of 0.5 was used to convert predicted probabilities into binary labels for ACC, REC, SPE, PRE, F1-score, and MCC. AUROC and AUPRC were calculated directly from continuous prediction probabilities and were therefore independent of this threshold. The same 0.5 threshold was used for the proteome-wide screening analysis.

Supplementary Methods S2. GAT, Bi-Mamba encoding, and gated fusion

For each protein, ESMFold-predicted C_α coordinates were used to construct a residue-level spatial proximity graph $G = (V, E)$. Each residue was connected to its 30 nearest spatial neighbors according to Euclidean distance. The resulting k-nearest-neighbor graph ($k = 30$) was symmetrized, self-loops were removed, and binary edge weights were used. The initial node features were the 256-dimensional ESM-2 residue embeddings:

$$H^{(0)} = [h_1^{(0)}, h_2^{(0)}, \ldots, h_L^{(0)}]. \tag{S7}$$

In the GAT branch, attention-based message passing was performed over the binary spatial graph. For residue i and its neighbor $j \in \mathcal{N}(i)$ at layer l, the unnormalized attention score was computed as

$$s_{ij}^{(l)} = \text{LeakyReLU}\left(\mathbf{a}^\top \left[\mathbf{W}^{(l)} h_i^{(l)} \| \mathbf{W}^{(l)} h_j^{(l)}\right]\right). \tag{S8}$$

The attention coefficients were normalized over the neighborhood of residue i:

$$\alpha_{ij}^{(l)} = \frac{\exp(s_{ij}^{(l)})}{\sum_{r \in \mathcal{N}(i)} \exp(s_{ir}^{(l)})}. \tag{S9}$$

The node representation was updated by

$$h_i^{(l+1)} = \delta\left(\sum_{j \in \mathcal{N}(i)} \alpha_{ij}^{(l)} \mathbf{W}^{(l)} h_j^{(l)}\right). \tag{S10}$$

In parallel, the Bi-Mamba branch modeled bidirectional sequential dependencies along the protein chain. The ESM-2 residue embeddings were processed in forward and reversed residue order:

$$H_{\text{Mamba}}^{\rightarrow} = \text{Mamba}^{\rightarrow}(H^{(0)}), \quad H_{\text{Mamba,rev}}^{\leftarrow} = \text{Mamba}^{\leftarrow}(\text{Reverse}(H^{(0)})). \tag{S11}$$

The backward output was restored to the original residue order and combined with the forward output by element-wise addition:

$$H_{\text{Mamba}}^{\leftarrow} = \text{Reverse}(H_{\text{Mamba,rev}}^{\leftarrow}), \qquad H_{\text{Mamba}} = H_{\text{Mamba}}^{\rightarrow} + H_{\text{Mamba}}^{\leftarrow}. \tag{S12}$$

The residue-level sequential representation was denoted as $h_{\text{Mamba},i}$.

The GAT and Bi-Mamba outputs were integrated by residue-wise gated fusion. The two branch outputs were first projected into a shared hidden space:

$$\tilde{h}_{\text{GAT},i} = \phi(\mathbf{W}_{\text{GAT}} h_{\text{GAT},i} + b_{\text{GAT}}), \quad \tilde{h}_{\text{Mamba},i} = \phi(\mathbf{W}_{\text{Mamba}} h_{\text{Mamba},i} + b_{\text{Mamba}}). \tag{S13}$$

A gate vector was computed as

$$z_i = \sigma\left(\mathbf{W}_z[\tilde{h}_{\text{GAT},i} \| \tilde{h}_{\text{Mamba},i}] + b_z\right), \tag{S14}$$

and the fused residue-level representation was

$$h_{\text{fuse},i} = z_i \odot \tilde{h}_{\text{GAT},i} + (1 - z_i) \odot \tilde{h}_{\text{Mamba},i}. \tag{S15}$$

The fused residue representations were pooled and concatenated with the ProtT5-derived global embedding:

$$v_{\text{pro}} = \text{MLP}\left(\text{Pool}(H_{\text{fuse}}) \| t_{\text{ProtT5}}\right). \tag{S16}$$

The resulting vector v_{pro} was used as the final protein representation, containing both protein language-model and ESMFold-derived structural information.

Supplementary Methods S3. Implementation, reproducibility, and Stacking-NAM details

Residue-level contextual representations were generated with the ESM-2 checkpoint `esm2_t33_650M_UR50D`, using the output from layer 33. Protein structures were predicted with `facebook/esmfold_v1` using `max_recycles=4`. Global protein-sequence representations were generated with `Rostlab/prot_t5_xl_half_uniref50-enc`, yielding 1,024-dimensional protein-level features.

Protein sequences longer than 1,000 amino acids were truncated to the first 1,000 residues before ESMFold prediction and language-model tokenization. The tokenizer used truncation with a maximum length of 1,000 and no added special tokens. No sliding-window, overlapping-segment, or segment-merging procedure was used for protein sequences. All 7,168 proteins in Dataset A and all 7,152 proteins in Dataset B were successfully processed by ESMFold; no sample was removed, replaced, or reassigned because of structure-prediction failure. For CDS-derived local heterogeneity features, a sliding window of 128 bp with a step size of 128 bp was used to calculate local GC content and Shannon entropy.

Residue-level graphs were constructed from ESMFold-predicted C_α coordinates as symmetrized 30-nearest-neighbor graphs. Self-loops were removed and binary edge weights were used. No radial-basis-function distance edge attributes were used in the final graph implementation. The GAT branch used four attention heads. The Bi-Mamba branch contained six layers with state dimension 16; the forward and backward Mamba modules were independent, and the backward output was restored to the original residue order before element-wise addition with the forward output.

The graph model was trained for 50 epochs using Adam with batch size 4, an initial learning rate of 9×10^{-5}, and zero weight decay. A cosine learning-rate schedule was used with five warm-up epochs and a minimum learning rate of 5×10^{-7}. Gradient accumulation was set to one and gradient clipping was not applied.

The Stacking-NAM framework integrated Random Forest (RF), RBF-kernel support vector machine (SVM), XGBoost, and multilayer perceptron (MLP) base learners. The key hyperparameters of the four base learners and the NAM meta-learner are summarized in Supplementary Table S13. Five-fold stratified out-of-fold probabilities were generated with `StratifiedKFold` (`shuffle=True`, `random_state=42`). Each development sample was therefore predicted by base learners that had not been fitted on that sample. The resulting four out-of-fold probabilities were used as NAM meta-level inputs. After out-of-fold feature generation, the four base learners were refitted on the complete development set and applied to the independent test set.

The protein-only model reported in the modality-ablation analysis and the GAT + Bi-Mamba + ProtT5 model reported in the protein-architecture ablation used different downstream prediction procedures. The protein-only result in Supplementary Table S4 and the corresponding main-text benchmark was obtained by supplying the complete protein representation to the four base learners followed by Stacking-NAM integration. In contrast, the GAT + Bi-Mamba + ProtT5 architecture-ablation result was obtained using direct classification from the protein encoder without the downstream Stacking-NAM ensemble. This difference in the prediction pipeline accounts for the Dataset B ACC values of 0.8681 (86.81%) and 0.8585 (85.85%), respectively; the two values therefore do not represent inconsistent evaluations of the same model configuration.

The global random seed was set to 42 for Python, NumPy, PyTorch, CUDA, data

splitting, and out-of-fold cross-validation. Experiments used Python 3.10.18, PyTorch 2.1.0 with CUDA 11.8, Transformers 4.30.0, scikit-learn 1.3.2, SciPy 1.10.1, and XGBoost 3.0.5. Hardware comprised an Intel Xeon Gold 6330 CPU and two NVIDIA GeForce RTX 4090 GPUs, each with approximately 24 GB of GPU memory.

Computational efficiency was evaluated on the 1,152-sample Dataset B independent test set. Complete preprocessing and inference required 16,496.54 s: ESMFold structure prediction, 16,275.65 s; ProtT5 embedding generation, 126.01 s; ESM-2 plus Graph-Mamba feature extraction, 55.30 s; CDS feature extraction, 37.00 s; and NAM inference, 2.58 s. Peak GPU memory during ESMFold/Graph-Mamba processing was 2.82 GB and peak CPU RAM was 5.30 GB; NAM inference used 9.34 MB of GPU memory and 834 MB of CPU RAM. These measurements show that end-to-end runtime was dominated by ESMFold structure prediction; accordingly, the linear sequence-length scaling discussed for Mamba applies to the sequence-modeling component rather than to the complete DeepGVS preprocessing and inference pipeline.

Supplementary Methods S4. Taxonomic composition and potential confounding analysis

Taxonomic annotations were reconstructed retrospectively for the curated samples at family, genus, and species levels. Taxonomic annotation was used only for post hoc confounding analyses and was not used as an input to DeepGVS. The CDS records used as model inputs and the retrospective taxonomic metadata were treated as separate data layers; lack of a uniquely recoverable current species assignment did not invalidate an otherwise traceable protein–CDS pair.

For each dataset, family-, genus-, and species-level composition was tabulated separately for VF and non-VF samples in the training, validation, and independent test splits. Complete split-by-label compositions are supplied as machine-readable Supplementary Data S1–S3, and annotation coverage is summarized in Table S8.

To quantify predictive information contained in taxonomy alone, categorical family, genus, and species identities were one-hot encoded and used as inputs to L2-regularized logistic-regression classifiers. Family-only, genus-only, species-only, and combined family+genus+species models were fitted on the training split. The regularization parameter was selected using only the validation split; the independent test split was not used for fitting or model selection. Unknown or unassigned categories were represented as unknown levels without using test labels.

Within-taxon sensitivity analyses were performed on the independent test set. A taxon was eligible only when it contained at least 10 VFs and 10 non-VFs, ensuring that AUROC was estimable within the stratum. These analyses were interpreted as sensitivity analyses rather than evidence of taxonomy independence, because strain- and lineage-level structure can remain within a species.

For direct comparison of the strongest combined taxonomy-only baseline with DeepGVS on the same test samples, paired stratified bootstrap resampling was used to quantify uncertainty in the AUROC and AUPRC differences. Positive and negative samples were resampled separately with replacement, and both models were evaluated on each identical resample.

Supplementary Methods S5. Two-level SHAP analysis, feature mapping, and modality aggregation

SHAP analyses were performed on the already trained models and evaluated on the held-out independent test sets. Interpretation was organized at two levels so that the base learner used for feature-level analysis was selected by an explicit ensemble-level criterion rather than by inspection of its feature SHAP profile.

Level 1: NAM-level learner attribution. For each feature configuration (CDS-only, protein-only, or fused), the Stacking-NAM meta-learner receives four meta-features: the positive-class probabilities ($p1$) from Random Forest, SVM-RBF, XGBoost, and MLP. SHAP was first applied to these four NAM inputs. The mean absolute SHAP value of each base-learner probability on the NAM logit was used to rank the four learners. The learner with the largest NAM-level contribution was designated as the primary learner for the corresponding feature-level interpretation. This rule selected SVM-RBF for the Dataset A fused model (Supplementary Fig. S6), Random Forest for the Dataset B fused model (Supplementary Fig. S13), XGBoost for the CDS-only models in Dataset A and Dataset B (Supplementary Figs. S9 and S17), and Random Forest for the protein-only models in Dataset A and Dataset B (Supplementary Figs. S11 and S19).

Level 2: feature-level attribution within the selected learner. SHAP was then calculated for the original input features of the selected base learner. The resulting primary feature-level analyses are shown for Dataset A fused SVM-RBF (main-text Figure 2 and Supplementary Fig. S7), Dataset A CDS-only XGBoost (Supplementary Fig. S10), Dataset A protein-only Random Forest (Supplementary Fig. S12), Dataset B fused Random Forest (Supplementary Fig. S14), Dataset B CDS-only XGBoost (Supplementary Fig. S18), and Dataset B protein-only Random Forest (Supplementary Fig. S20). The CDS-only and protein-only models were separately trained unimodal predictors; their SHAP plots characterize feature importance within those predictors and are not decompositions of the fused DeepGVS prediction. Absolute SHAP magnitudes from separately trained unimodal models were therefore not compared quantitatively to infer cross-model modality contributions.

Sensitivity analyses. Fused-model MLP SHAP analyses were retained as learner-sensitivity analyses for Dataset A (Supplementary Fig. S8) and Dataset B (Supplementary Fig. S16). For the selected Dataset B Random Forest fused learner, a CDS-restricted ranking is additionally shown in Supplementary Fig. S15 to identify the highest-ranked CDS-derived variables even when they do not appear among the globally top-ranked fused features.

Let $\phi_{i,j}$ denote the SHAP value for sample i and input feature j. The global importance of feature j was defined as its mean absolute SHAP value across all N independent-test samples:

$$I_j = \frac{1}{N}\sum_{i=1}^{N} |\phi_{i,j}|. \tag{S17}$$

Individual-feature bar plots display the top-ranked features according to I_j. CDS-derived coordinates were mapped back to their original sequence descriptors, including explicit nucleotide k-mer and other CDS-derived variables. Learned Graph-Mamba coordinates were labeled "GM latent" and were not mapped one-to-one to amino-acid residues, structural regions, or molecular mechanisms.

For fused models, the modality donut was calculated across *all* fused input features, not only the displayed top-ranked features. Feature-wise mean absolute SHAP values

were summed separately over the complete CDS and GM feature groups:

$$S_{\text{CDS}} = \sum_{j \in \text{CDS}} I_j, \qquad S_{\text{GM}} = \sum_{j \in \text{GM}} I_j. \tag{S18}$$

The two modality totals were then normalized by their combined importance:

$$P_{\text{CDS}} = \frac{S_{\text{CDS}}}{S_{\text{CDS}} + S_{\text{GM}}} \times 100\%, \qquad P_{\text{GM}} = \frac{S_{\text{GM}}}{S_{\text{CDS}} + S_{\text{GM}}} \times 100\%. \tag{S19}$$

Thus, the donut represents importance-weighted modality contributions rather than the proportion of features in each modality or the composition of the displayed top-K features. The single-modality donuts in CDS-only or protein-only plots are necessarily 100% for the represented modality and are shown only as visual identifiers; they were not used for cross-model modality comparisons.

For the primary Dataset A SVM-RBF fused analysis, the global modality shares were 24.8% CDS and 75.2% GM (main-text Figure 2; Supplementary Fig. S7). The Dataset A MLP sensitivity analysis yielded 22.0% CDS and 78.0% GM (Supplementary Fig. S8). For the primary Dataset B Random Forest fused analysis, the corresponding shares were 3.3% CDS and 96.7% GM (Supplementary Fig. S14), whereas the Dataset B MLP sensitivity analysis yielded 20.7% CDS and 79.3% GM (Supplementary Fig. S16). These values were interpreted as learner-dependent predictive attributions rather than fixed biological modality weights.

Table S1: Class composition of the training, validation, and independent test sets used in DeepGVS.

Dataset	Split	VFs	Non-VFs	Total samples	Usage
Dataset A	Training set	2499	2500	4999	Model fitting
	Validation set	400	400	800	Model selection
	Independent test set	685	684	1369	Final evaluation
	Total	3584	3584	7168	–
Dataset B	Training set	2693	2707	5400	Model fitting
	Validation set	307	293	600	Model selection
	Independent test set	576	576	1152	Final evaluation
	Total	3576	3576	7152	–

Note: Dataset A was constructed from VFDB Core Set A and whole-proteome-derived non-VFs after homology reduction, CDS mapping, and class balancing. Dataset B retained the original DeepVF accession-level independent test set (576 VFs and 576 non-VFs). CDS retrieval was performed after the original protein-level partitions had been established and did not alter independent-test membership. The 6,000-sample balanced development set was divided into the training and validation splits shown above.

Table S2: Definitions, calculation rules, and dimensions of CDS-derived features used in DeepGVS.

Feature group	Symbol	Feature	Calculation rule	Dimension
Global statistical features	v_{basic}	Sequence length	Total number of nucleotides in the CDS, denoted as L	1
		GC content	$(N_G + N_C)/L$	1
		AT content	$(N_A + N_T)/L$	1
		Ambiguous nucleotide ratio	N_N/L	1
		Shannon entropy	$-\sum_{b\in\{A,C,G,T\}} p_b \log_2 p_b$	1
		Number of ORFs	Number of detected open reading frames in the CDS	1
		Maximum ORF length	Length of the longest detected open reading frame	1
k-mer frequency features	v_{kmer}	2-mer frequencies	Normalized frequencies of all possible nucleotide 2-mers	$4^2 = 16$
		3-mer frequencies	Normalized frequencies of all possible nucleotide 3-mers	$4^3 = 64$
		4-mer frequencies	Normalized frequencies of all possible nucleotide 4-mers	$4^4 = 256$
Local heterogeneity features	v_{window}	Mean window-level GC content	Mean GC content calculated across sliding windows	1
		Standard deviation of window-level GC content	Standard deviation of GC content calculated across sliding windows	1
		Mean window-level Shannon entropy	Mean Shannon entropy calculated across sliding windows	1
		Standard deviation of window-level Shannon entropy	Standard deviation of Shannon entropy calculated across sliding windows	1
Repeat and low-complexity features	v_{repeat}	Longest A run	Maximum length of consecutive A nucleotides	1
		Longest C run	Maximum length of consecutive C nucleotides	1
		Longest G run	Maximum length of consecutive G nucleotides	1
		Longest T run	Maximum length of consecutive T nucleotides	1
		Palindromic 4-mer ratio	Proportion of reverse-complement palindromic 4-mers	1
		Maximum dinucleotide tandem repeat count	Maximum number of consecutive dinucleotide tandem repeats	1
		Unique 4-mer ratio	Number of unique 4-mers divided by the total number of 4-mer positions	1
Total	v_{CDS}	Concatenated CDS representation	$v_{\text{basic}} \oplus v_{\text{kmer}} \oplus v_{\text{window}} \oplus v_{\text{repeat}}$	354

Note: Local heterogeneity features were calculated using a 128-bp window with a 128-bp step size.

Table S3: Five-fold cross-validation performance comparison of single-modality and bimodal models on Dataset A and Dataset B.

Dataset	Model	ACC	F1	REC	SPE	MCC	PRE
Dataset A	CDS-only	0.9215 ± 0.0170	0.9246 ± 0.0103	0.9621 ± 0.0137	0.8810 ± 0.0124	0.8459 ± 0.0216	0.8899 ± 0.0224
	Protein-only	0.9797 ± 0.0020	0.9797 ± 0.0020	0.9810 ± 0.0039	0.9783 ± 0.0020	0.9593 ± 0.0040	0.9784 ± 0.0056
	DeepGVS	**0.9860 ± 0.0040**	**0.9860 ± 0.0040**	**0.9869 ± 0.0038**	**0.9852 ± 0.0066**	**0.9721 ± 0.0080**	**0.9851 ± 0.0085**
Dataset B	CDS-only	0.7188 ± 0.0119	0.7212 ± 0.0142	0.7280 ± 0.0269	0.7097 ± 0.0249	0.4382 ± 0.0236	0.7145 ± 0.0381
	Protein-only	0.9823 ± 0.0021	0.9823 ± 0.0021	0.9813 ± 0.0039	0.9834 ± 0.0030	0.9647 ± 0.0043	0.9833 ± 0.0057
	DeepGVS	**0.9823 ± 0.0024**	**0.9823 ± 0.0025**	**0.9800 ± 0.0054**	**0.9845 ± 0.0022**	**0.9647 ± 0.0049**	**0.9846 ± 0.0074**

Note: Values are mean ± standard deviation across five folds. The folds were random stratified partitions of the development set and were not explicitly taxonomy-disjoint or sequence-cluster-disjoint; CD-HIT was not rerun within individual folds. Consequently, these internal cross-validation estimates should not be interpreted as equivalent to independent-test generalization. Protein-only denotes the complete protein representation, including protein language-model and ESMFold-derived structural information.

Table S4: Independent test performance comparison of single-modality and bimodal models on Dataset A and Dataset B.

Dataset	Model	ACC	SPE	MCC	AUROC	AUPRC	PRE	F1	REC
Dataset A	CDS-only	0.9254	0.8932	0.8527	0.9676	0.9538	0.8998	**0.9278**	**0.9576**
	Protein-only	0.8970	0.9050	0.7941	0.9541	0.9466	0.9036	0.8962	0.8891
	DeepGVS	**0.9262**	**0.9473**	**0.8532**	**0.9795**	**0.9766**	**0.9451**	0.9246	0.9051
Dataset B	CDS-only	0.7274	0.7587	0.4558	0.8133	0.8100	0.7426	0.7186	0.6962
	Protein-only	0.8681	0.8941	0.7371	0.9269	0.9345	0.8883	0.8645	0.8420
	DeepGVS	**0.8750**	**0.9010**	**0.7510**	**0.9275**	**0.9365**	**0.8956**	**0.8717**	**0.8490**

Table S5: Five-fold cross-validation performance comparison of base classifiers under different feature configurations on Dataset A and Dataset B.

Dataset	Feature configuration	Classifier	ACC	F1	MCC	AUROC	AUPRC
Dataset A	CDS-only	RF	0.9090 ± 0.0060	0.9134 ± 0.0056	0.8224 ± 0.0118	0.9548 ± 0.0062	0.9354 ± 0.0103
		SVM	0.8886 ± 0.0108	0.8905 ± 0.0105	0.7779 ± 0.0215	0.9483 ± 0.0043	0.9312 ± 0.0078
		XGBoost	0.9176 ± 0.0077	0.9207 ± 0.0076	0.8379 ± 0.0159	0.9613 ± 0.0049	0.9456 ± 0.0077
		MLP	0.8808 ± 0.0093	0.8832 ± 0.0091	0.7625 ± 0.0184	0.9382 ± 0.0066	0.9140 ± 0.0103
	Protein-only	RF	0.9797 ± 0.0021	0.9797 ± 0.0021	0.9593 ± 0.0042	0.9959 ± 0.0026	0.9946 ± 0.0051
		SVM	0.9791 ± 0.0024	0.9791 ± 0.0024	0.9583 ± 0.0048	0.9958 ± 0.0024	0.9953 ± 0.0034
		XGBoost	0.9793 ± 0.0019	0.9794 ± 0.0019	0.9587 ± 0.0038	0.9963 ± 0.0021	0.9952 ± 0.0039
		MLP	0.9764 ± 0.0040	0.9765 ± 0.0039	0.9528 ± 0.0079	0.9957 ± 0.0018	0.9948 ± 0.0038
	DeepGVS	RF	0.9747 ± 0.0044	0.9746 ± 0.0044	0.9493 ± 0.0088	0.9961 ± 0.0010	0.9952 ± 0.0015
		SVM	$\mathbf{0.9826 \pm 0.0048}$	$\mathbf{0.9826 \pm 0.0048}$	$\mathbf{0.9652 \pm 0.0097}$	$\mathbf{0.9976 \pm 0.0016}$	0.9968 ± 0.0025
		XGBoost	0.9793 ± 0.0040	0.9793 ± 0.0040	0.9586 ± 0.0079	0.9975 ± 0.0012	$\mathbf{0.9970 \pm 0.0019}$
		MLP	0.9791 ± 0.0044	0.9792 ± 0.0044	0.9583 ± 0.0089	0.9970 ± 0.0014	0.9961 ± 0.0021
Dataset B	CDS-only	RF	0.6933 ± 0.0069	0.6887 ± 0.0088	0.3870 ± 0.0138	0.7685 ± 0.0095	0.7629 ± 0.0145
		SVM	0.6990 ± 0.0104	0.6971 ± 0.0116	0.3981 ± 0.0208	0.7753 ± 0.0098	0.7744 ± 0.0127
		XGBoost	$\mathbf{0.7165 \pm 0.0094}$	$\mathbf{0.7139 \pm 0.0123}$	$\mathbf{0.4333 \pm 0.0185}$	$\mathbf{0.7976 \pm 0.0109}$	$\mathbf{0.8010 \pm 0.0147}$
		MLP	0.6833 ± 0.0084	0.6774 ± 0.0228	0.3686 ± 0.0156	0.7567 ± 0.0110	0.7530 ± 0.0121
	Protein-only	RF	0.9792 ± 0.0053	0.9791 ± 0.0054	0.9584 ± 0.0106	0.9944 ± 0.0012	0.9952 ± 0.0011
		SVM	0.9812 ± 0.0034	0.9812 ± 0.0035	0.9624 ± 0.0069	0.9949 ± 0.0005	0.9953 ± 0.0009
		XGBoost	$\mathbf{0.9817 \pm 0.0035}$	$\mathbf{0.9816 \pm 0.0036}$	$\mathbf{0.9634 \pm 0.0070}$	$\mathbf{0.9952 \pm 0.0009}$	$\mathbf{0.9955 \pm 0.0009}$
		MLP	0.9807 ± 0.0030	0.9807 ± 0.0030	0.9614 ± 0.0061	0.9946 ± 0.0018	$\mathbf{0.9955 \pm 0.0014}$
	DeepGVS	RF	0.9793 ± 0.0048	0.9793 ± 0.0049	0.9587 ± 0.0096	0.9943 ± 0.0010	0.9946 ± 0.0017
		SVM	$\mathbf{0.9815 \pm 0.0031}$	$\mathbf{0.9815 \pm 0.0032}$	$\mathbf{0.9630 \pm 0.0063}$	$\mathbf{0.9951 \pm 0.0006}$	0.9956 ± 0.0008
		XGBoost	0.9813 ± 0.0040	0.9813 ± 0.0040	0.9627 ± 0.0079	0.9950 ± 0.0009	0.9955 ± 0.0011
		MLP	0.9812 ± 0.0031	0.9811 ± 0.0032	0.9624 ± 0.0061	$\mathbf{0.9951 \pm 0.0015}$	$\mathbf{0.9960 \pm 0.0013}$

Note: Protein-only denotes the complete protein representation, including protein language-model and ESMFold-derived structural information. Values are mean $\pm$ standard deviation across five folds.

Table S6: Independent test performance comparison of base classifiers and Stacking-NAM under different feature configurations on Dataset A and Dataset B.

Dataset	Feature configuration	Classifier	ACC	F1	REC	SPE	MCC	PRE
Dataset A	CDS-only	RF	0.9131	0.9167	0.9562	0.8699	0.8292	0.8804
		SVM	0.8875	0.8876	0.8876	0.8874	0.7750	0.8876
		XGBoost	0.9204	0.9227	0.9504	0.8904	0.8423	0.8967
		MLP	0.8795	0.8805	0.8876	0.8713	0.7590	0.8736
		Stacking-NAM	**0.9254**	**0.9279**	**0.9577**	**0.8933**	**0.8528**	**0.8999**
	Protein-only	RF	0.8948	0.8941	0.8876	0.9020	0.7897	0.9007
		SVM	0.8948	0.8932	0.8788	**0.9108**	0.7900	**0.9080**
		XGBoost	0.8955	0.8945	0.8847	0.9064	0.7913	0.9045
		MLP	0.8912	0.8913	**0.8920**	0.8904	0.7823	0.8907
		Stacking-NAM	**0.8970**	**0.8962**	0.8891	0.9050	**0.7941**	0.9036
	DeepGVS	RF	0.9109	0.9092	0.8920	0.9298	0.8224	0.9272
		SVM	**0.9277**	**0.9260**	0.9036	**0.9518**	**0.8564**	**0.9494**
		XGBoost	0.9080	0.9067	0.8934	0.9225	0.8163	0.9203
		MLP	0.9160	0.9144	0.8964	0.9357	0.8326	0.9331
		Stacking-NAM	0.9262	0.9247	**0.9051**	0.9474	0.8532	0.9451
Dataset B	CDS-only	RF	0.6988	0.6926	0.6788	0.7188	0.3979	0.7071
		SVM	**0.7335**	**0.7300**	**0.7205**	0.7465	**0.4672**	**0.7398**
		XGBoost	0.7161	0.7104	0.6962	0.7361	0.4326	0.7251
		MLP	0.6962	0.6869	0.6667	0.7257	0.3930	0.7085
		Stacking-NAM	0.7274	0.7186	0.6962	**0.7587**	0.4558	0.7426
	Protein-only	RF	0.8490	0.8468	0.8351	0.8628	0.6982	0.8589
		SVM	0.8611	0.8571	0.8333	0.8889	0.7233	**0.8824**
		XGBoost	0.8576	0.8536	0.8299	0.8854	0.7164	0.8787
		MLP	0.8542	0.8489	0.8194	0.8889	0.7100	0.8806
		Stacking-NAM	**0.8681**	**0.8645**	**0.8420**	**0.8941**	**0.7371**	0.8883
	DeepGVS	RF	0.8490	0.8468	0.8351	0.8628	0.6982	0.8589
		SVM	0.8646	0.8607	0.8368	0.8924	0.7303	0.8860
		XGBoost	0.8611	0.8569	0.8316	0.8906	0.7235	0.8838
		MLP	0.8655	0.8617	0.8385	0.8924	0.7320	0.8862
		Stacking-NAM	**0.8750**	**0.8717**	**0.8490**	**0.9010**	**0.7510**	**0.8956**

Note: Protein-only
denotes the complete protein sequence–structure representation, including protein language-model features and ESMFold-derived structural information. For Dataset B, the Protein-only RF and DeepGVS RF used 1,792 and 2,146 input features, respectively, and the two rows are not duplicated entries. At the fixed decision threshold of 0.50, their binary prediction vectors differed for four of the 1,152 independent-test samples. However, these changes resulted in identical confusion-matrix counts (TP = 481, TN = 497, FP = 79, and FN = 95), and therefore identical threshold-dependent metrics. Their continuous probability outputs were different: Protein-only RF, AUROC = 0.9208, AUPRC = 0.9277, LogLoss = 0.4459; DeepGVS RF, AUROC = 0.9198, AUPRC = 0.9298, LogLoss = 0.4226.

Table S7: eggNOG-based annotation results for seven of the eight high-confidence candidate proteins identified by proteome-wide screening of *Escherichia coli* O157:H7.

Protein ID	Seed ortholog	E-value	Score	COG	Description	Name	PFAMs
Q8X718_ECO57	155864.EDL933_1811	0.0	995.0	L	Recombinase activity	ybcK	Recombinase; Resolvase; Zn_ribbon_recom
A0A0H3JN46_ECO57	502347.ESCAB7627_2445	7.72×10^{-159}	447.0	–	–	–	–
Q8X7K5_ECO57	155864.EDL933_0293	2.00×10^{-148}	417.0	–	–	–	–
A0A0H3JF05_ECO57	155864.EDL933_1019	0.0	1206.0	–	–	–	–
A0A0H3JMF4_ECO57	316407.85676800	8.06×10^{-131}	378.0	S	Protein of unknown function (DUF2713)	yjbM	DUF2713
Q8X5D5_ECO57	199310.c1459	3.72×10^{-86}	254.0	S	Phage tail assembly protein T	–	Phage_tail_T
Q8X7K4_ECO57	1115512.EH105704_04_01440	1.17×10^{-42}	145.0	–	–	–	–

Table S8: Taxonomic annotation coverage in Datasets A and B.

Dataset	Family	Genus	Species
Dataset A	98.5%	96.9%	86.2%
Dataset B	97.7%	96.5%	96.1%

Complete taxonomic compositions for VF and non-VF samples in each training, validation, and independent test split are provided in Supplementary Data S1–S3.

Table S9: Independent-test performance of taxonomy-only baselines compared with unimodal models and DeepGVS.

Dataset	Model/input	AUROC	AUPRC
Dataset A	Family-only taxonomy	0.820	0.817
	Genus-only taxonomy	0.852	0.841
	Species-only taxonomy	0.922	0.906
	Family+genus+species taxonomy	0.933	0.930
	CDS-only	0.9676	0.9538
	Protein-only	0.9541	0.9466
	DeepGVS	0.9795	0.9766
Dataset B	Family-only taxonomy	0.775	0.775
	Genus-only taxonomy	0.807	0.803
	Species-only taxonomy	0.866	0.853
	Family+genus+species taxonomy	0.868	0.855
	CDS-only	0.8133	0.8100
	Protein-only	0.9269	0.9345
	DeepGVS	0.9275	0.9365

Table S10: Summary of taxonomically stratified independent-test sensitivity analyses. Eligibility required at least 10 VFs and 10 non-VFs within each taxon.

Dataset	Rank	Eligible taxa	Within-taxon AUROC range
Dataset A	Family	8	0.946–1.000
	Genus	10	0.939–1.000
	Species	7	0.935–1.000
Dataset B	Family	14	
	Genus	14	0.799–0.991 across eligible strata
	Species	9	

Complete taxon-level sample counts and metrics are provided in Supplementary Data S4. These are sensitivity analyses and do not exclude within-species strain or lineage confounding.

Table S11: Dataset A within-species independent-test sensitivity results for species meeting the minimum sample criterion.

Species	Non-VFs	VFs	AUROC	AUPRC
Salmonella enterica	24	25	0.935	0.887
Vibrio cholerae	17	15	0.961	0.953
Yersinia enterocolitica	16	10	0.969	0.943
Mycobacterium tuberculosis	11	29	0.978	0.992
Escherichia coli	217	20	0.982	0.914
Pseudomonas aeruginosa	31	58	0.988	0.994
Vibrio parahaemolyticus	16	10	1.000	1.000

Table S12: Paired stratified-bootstrap comparison of the combined taxonomy-only model and DeepGVS on the same independent-test samples.

Dataset	Quantity	Estimate	95% bootstrap CI
Dataset A	DeepGVS AUROC	0.980	0.973–0.985
	Taxonomy-only AUROC	0.933	0.920–0.944
	AUROC difference	0.047	0.035–0.060
	DeepGVS AUPRC	0.977	0.967–0.985
	Taxonomy-only AUPRC	0.930	0.916–0.942
	AUPRC difference	0.047	0.032–0.062
Dataset B	DeepGVS AUROC	0.927	0.912–0.942
	Taxonomy-only AUROC	0.868	0.848–0.888
	AUROC difference	0.059	0.036–0.084
	DeepGVS AUPRC	0.936	0.923–0.950
	Taxonomy-only AUPRC	0.855	0.832–0.877
	AUPRC difference	0.082	0.057–0.107

Table S13: Implementation, model hyperparameters, software, hardware, runtime, and memory summary.

Item	Configuration/result
ESM-2	`esm2_t33_650M_UR50D`; layer 33
ESMFold	`facebook/esmfold_v1`; `max_recycles=4`
ProtT5	`Rostlab/prot_t5_xl_half_uniref50-enc`; 1,024-dimensional global representation
Length handling	First 1,000 residues retained; no sliding-window, overlapping-segment, or segment-merging procedure for protein sequences
ESMFold processing	All 7,168 Dataset A proteins and all 7,152 Dataset B proteins were processed successfully; no samples were removed, replaced, or reassigned because of structure-prediction failure
Graph construction	Symmetrized 30-NN graph from C_α coordinates; no self-loops; binary edge weights; no RBF distance edge attributes
GAT	4 attention heads
Bi-Mamba	6 layers; state dimension 16; independent forward/backward modules; aligned outputs combined by element-wise addition
Graph-model training	50 epochs; Adam; batch size 4; learning rate 9×10^{-5}; weight decay 0; cosine schedule; 5 warm-up epochs; minimum learning rate 5×10^{-7}; gradient accumulation 1; no gradient clipping
Random Forest	`n_estimators=500`
RBF-SVM	`C=4.0`; `gamma="scale"`
XGBoost	`n_estimators=800`; `max_depth=8`; `learning_rate=0.03`
MLP	`hidden_layer_sizes=(512, 256)`
Stacking procedure	5-fold stratified OOF probabilities; `shuffle=True`; `random_state=42`; base learners refitted on the complete development set before independent-test prediction
NAM meta-learner	Hidden dimension 8; one feature-subnetwork layer; no dropout; learning rate 1×10^{-3}; weight decay 1×10^{-3}; batch size 256; maximum 200 epochs; early-stopping patience 10
Random seed	42 for Python, NumPy, PyTorch, CUDA, data splitting, and OOF cross-validation
Software	Python 3.10.18; PyTorch 2.1.0/CUDA 11.8; Transformers 4.30.0; scikit-learn 1.3.2; SciPy 1.10.1; XGBoost 3.0.5
Hardware	Intel Xeon Gold 6330; two NVIDIA RTX 4090 GPUs (approximately 24 GB each)
Dataset B independent-test pipeline runtime ($n = 1{,}152$)	16,496.54 s total; ESMFold 16,275.65 s; ProtT5 126.01 s; ESM-2 + Graph-Mamba 55.30 s; CDS 37.00 s; NAM 2.58 s
Peak memory	2.82 GB GPU and 5.30 GB CPU RAM during ESMFold/Graph-Mamba; NAM 9.34 MB GPU and 834 MB CPU RAM

Table S14: Global SHAP modality aggregation across all fused input dimensions for primary and sensitivity base learners.

Dataset	Base learner / role	CDS share	GM/protein share
Dataset A	SVM-RBF, primary fused interpretation	24.8%	75.2%
Dataset A	MLP, sensitivity analysis	22.0%	78.0%
Dataset B	RF, primary fused interpretation	3.3%	96.7%
Dataset B	MLP, sensitivity analysis	20.7%	79.3%

Shares were computed by summing feature-wise mean absolute SHAP importance over all dimensions within each modality and normalizing the two modality totals to 100%. They are not top-K-only percentages. Primary base learners were identified by the largest NAM meta-feature mean absolute SHAP contribution for the corresponding feature configuration.

Supplementary Figures

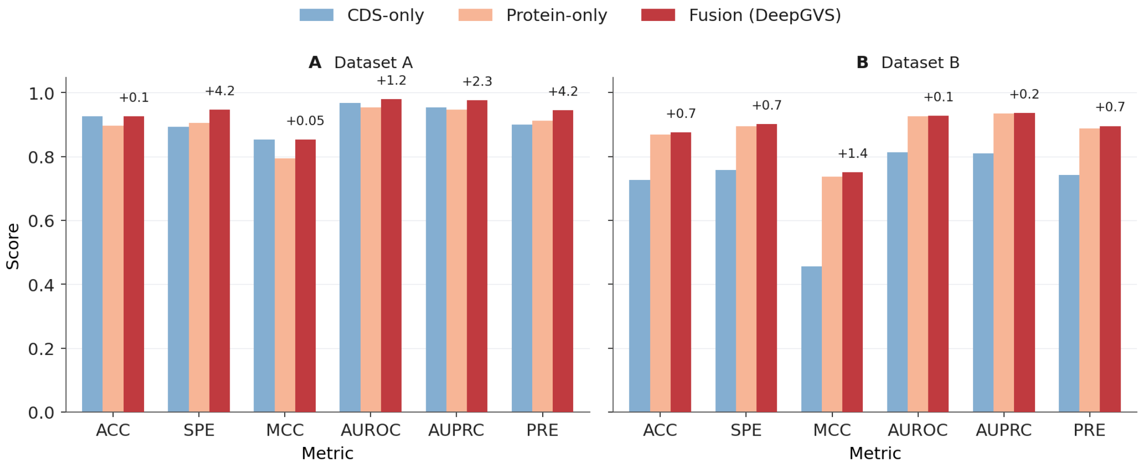

Figure S1: Independent test performance of ablation models. (a) Dataset A. (b) Dataset B. Values above bars report the observed metric differences between DeepGVS and the stronger single-modality baseline; the magnitude and direction of the incremental change are dataset- and metric-dependent. ROC and PRC denote AUROC and AUPRC, respectively.

Alt text: Bar charts compare CDS-only, protein-only, and fused DeepGVS test metrics for Datasets A and B, showing generally modest and metric-dependent changes after fusion.

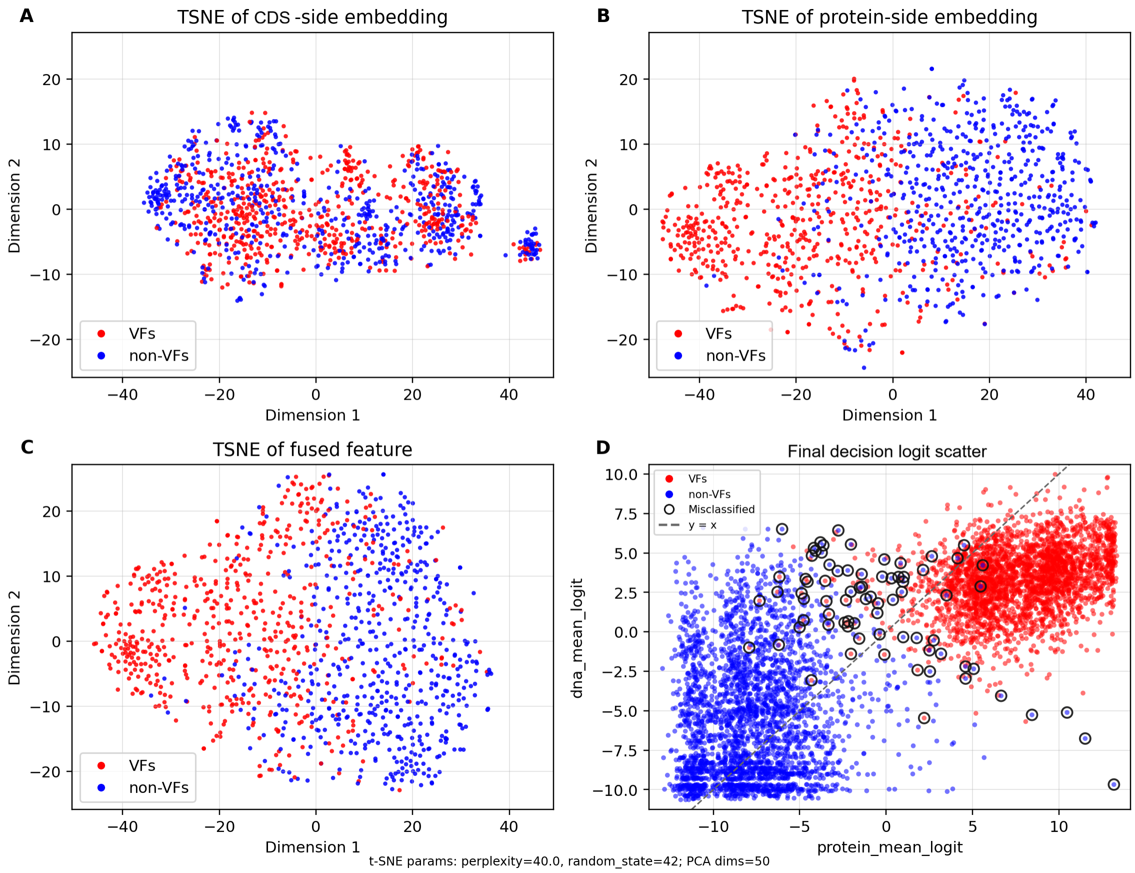

Figure S2: Feature-space visualization and decision-level comparison on Dataset A. (A–C) t-SNE visualizations of CDS-side, protein-side, and fused representations. (D) Decision-level logit scatter plot comparing protein-only and CDS-only models.

Alt text: Dataset A t-SNE plots for CDS, protein, and fused representations plus a scatter plot comparing CDS-only and protein-only decision logits.

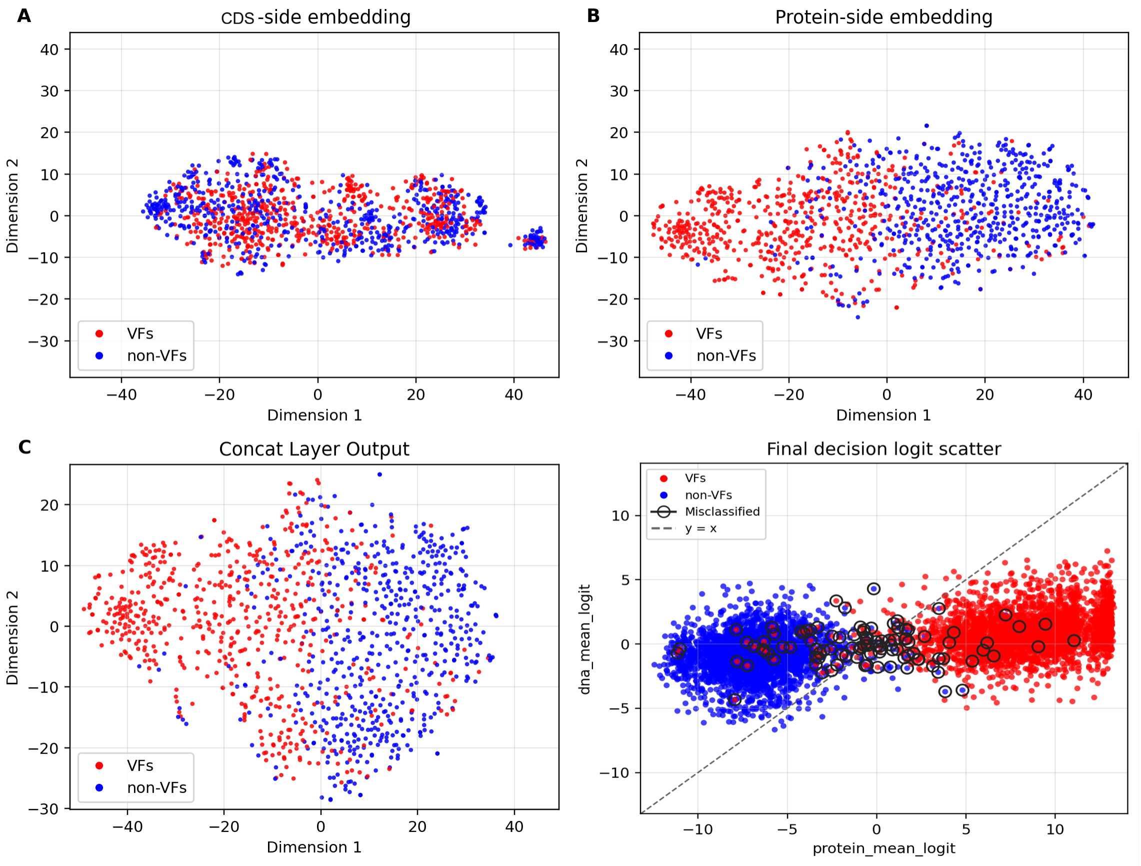

Figure S3: Feature-space visualization and decision-level comparison on Dataset B. (A–C) t-SNE visualizations of CDS-side, protein-side, and fused representations. (D) Decision-level logit scatter plot comparing protein-only and CDS-only models.

Alt text: Dataset B t-SNE plots for CDS, protein, and fused representations plus a scatter plot comparing CDS-only and protein-only decision logits.

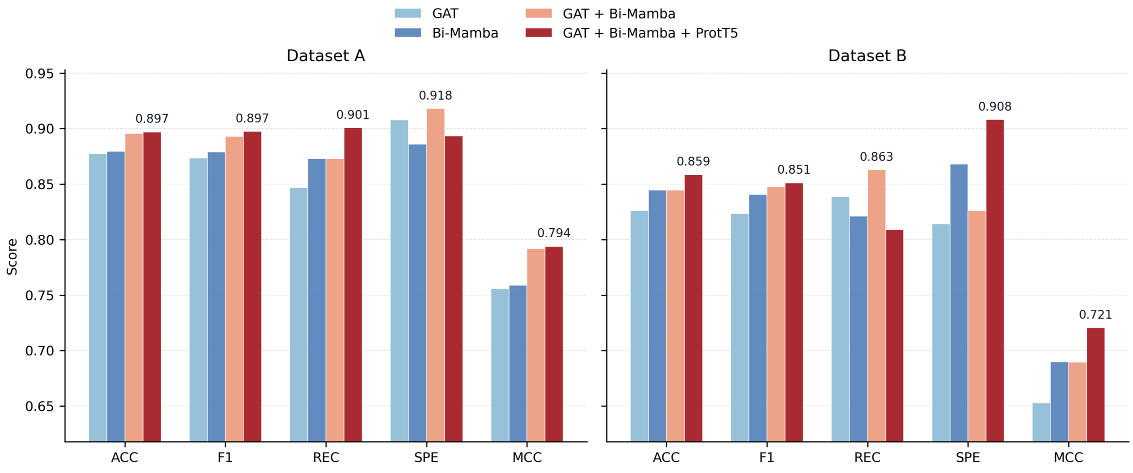

Figure S4: Performance of protein-modality architectures on Dataset A and Dataset B, including GAT, Bi-Mamba, GAT + Bi-Mamba, and GAT + Bi-Mamba + ProtT5.

Alt text: Bar charts compare four protein-branch architectures across evaluation metrics in Datasets A and B.

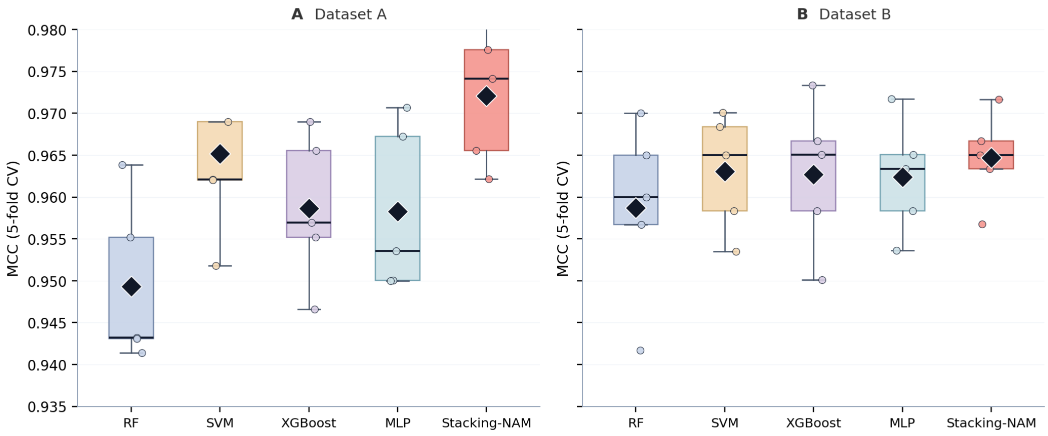

Figure S5: Five-fold cross-validation MCC distributions of RF, SVM, XGBoost, MLP, and Stacking-NAM on Dataset A and Dataset B. Boxes indicate interquartile ranges, center lines indicate medians, black diamonds indicate means, and points indicate fold-level results.

Alt text: Box plots show fold-level MCC distributions for four base classifiers and Stacking-NAM across both datasets.

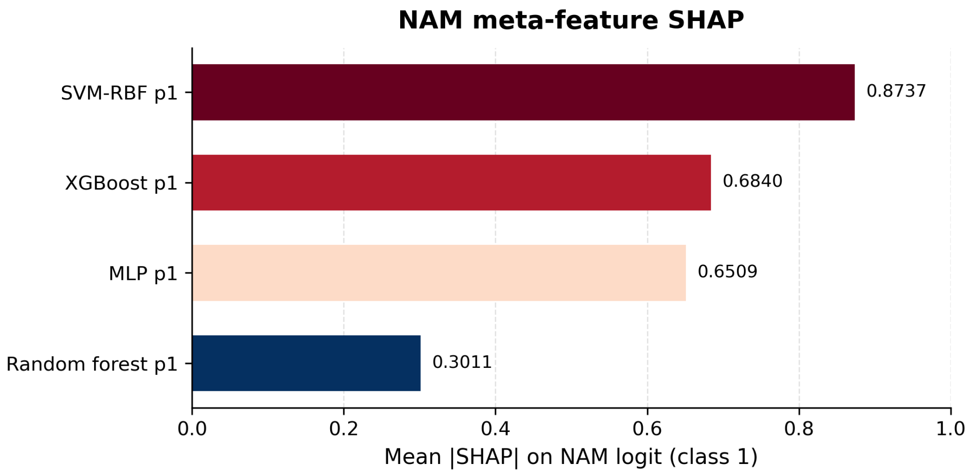

Figure S6: NAM meta-feature SHAP for the Dataset A fused stacking model. SVM-RBF has the largest mean absolute contribution to the NAM logit and was therefore selected for the primary feature-level analysis.

Alt text: Dataset A fused NAM meta-feature SHAP ranks the four base-learner probabilities, with SVM-RBF highest.

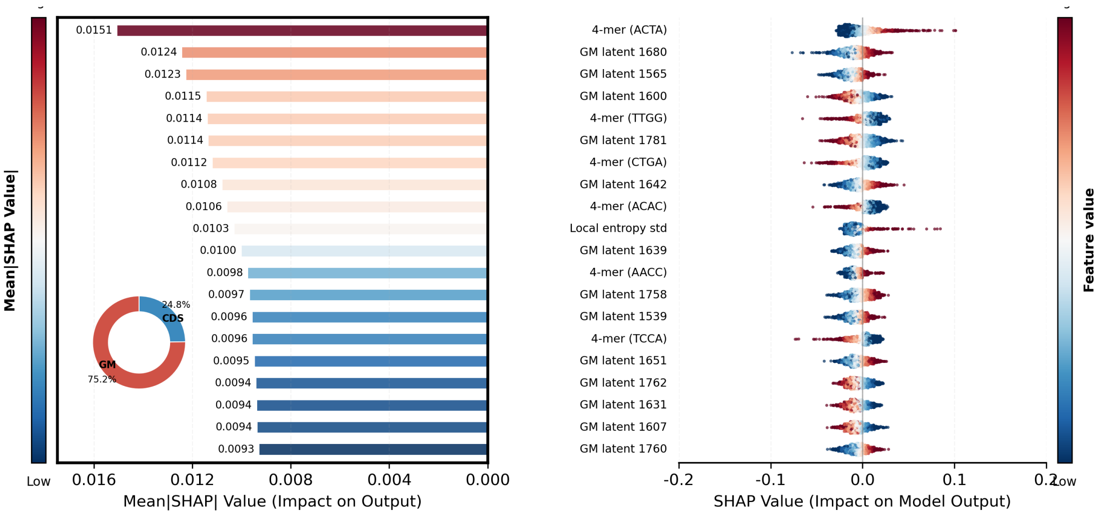

Figure S7: Primary feature-level SHAP analysis for the Dataset A fused model using the SVM-RBF learner selected by the preceding NAM-level analysis. The global modality donut reports CDS 24.8% and GM 75.2%.

Alt text: Dataset A fused SVM-RBF SHAP plot containing CDS descriptors and GM latent dimensions; the donut reports 24.8% CDS and 75.2% GM.

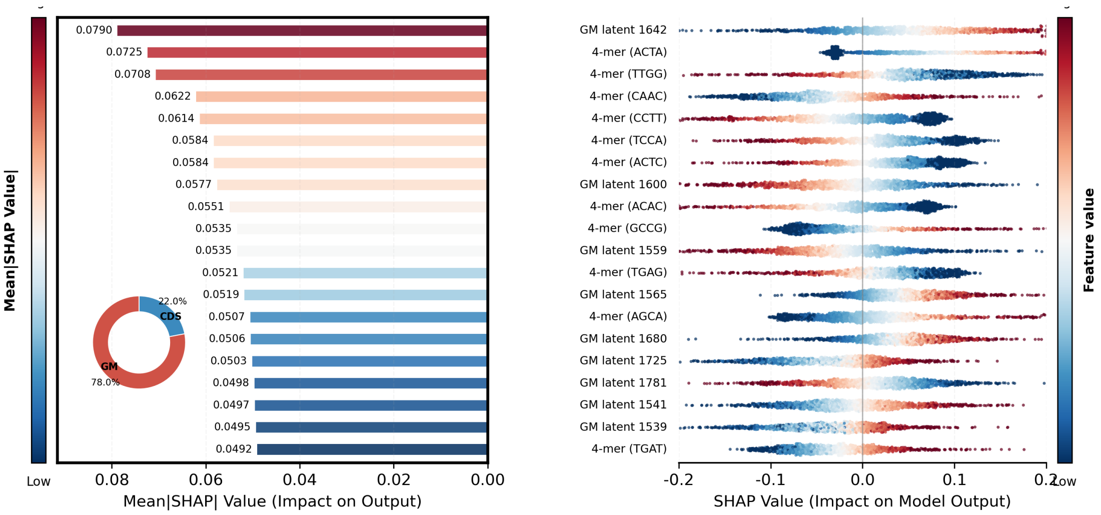

Figure S8: Fused-model SHAP sensitivity analysis for the Dataset A MLP learner. The top-ranked features include explicit CDS descriptors and GM latent dimensions; the global modality donut reports CDS 22.0% and GM 78.0%.

Alt text: Dataset A fused MLP SHAP sensitivity plot showing CDS descriptors and GM latent dimensions; the donut reports 22.0% CDS and 78.0% GM.

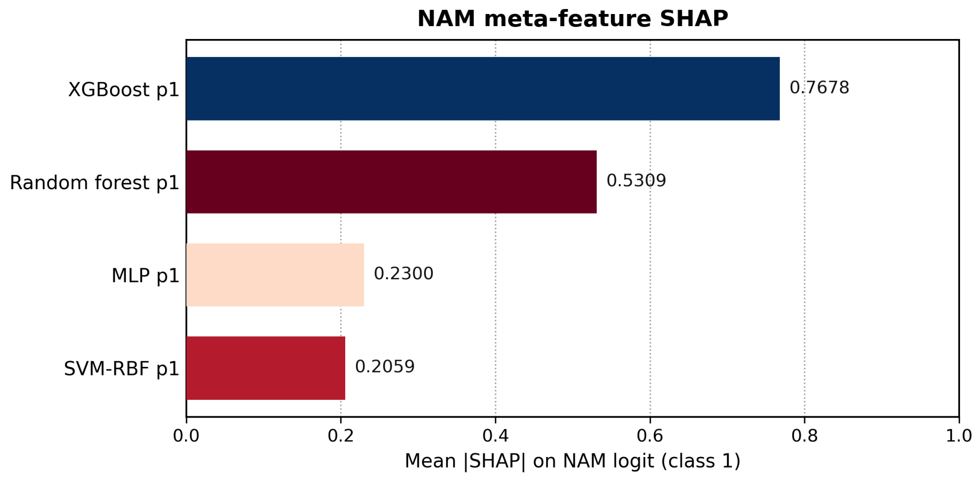

Figure S9: NAM meta-feature SHAP for the separately trained Dataset A CDS-only stacking model. XGBoost has the largest mean absolute contribution and was therefore selected for the corresponding feature-level SHAP analysis.

Alt text: Dataset A CDS-only NAM meta-feature SHAP ranks the four base learners, with XGBoost highest.

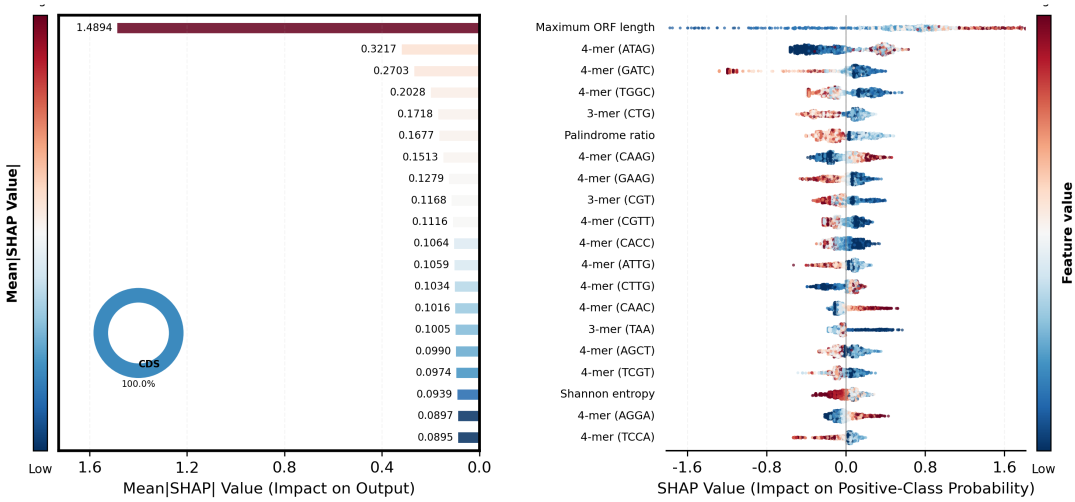

Figure S10: SHAP analysis of the separately trained Dataset A CDS-only XGBoost model selected by the preceding NAM-level analysis. CDS variables are labeled with their original sequence descriptors.

Alt text: Dataset A CDS-only XGBoost SHAP plot ranking explicit nucleotide-derived variables and showing feature-level effects.

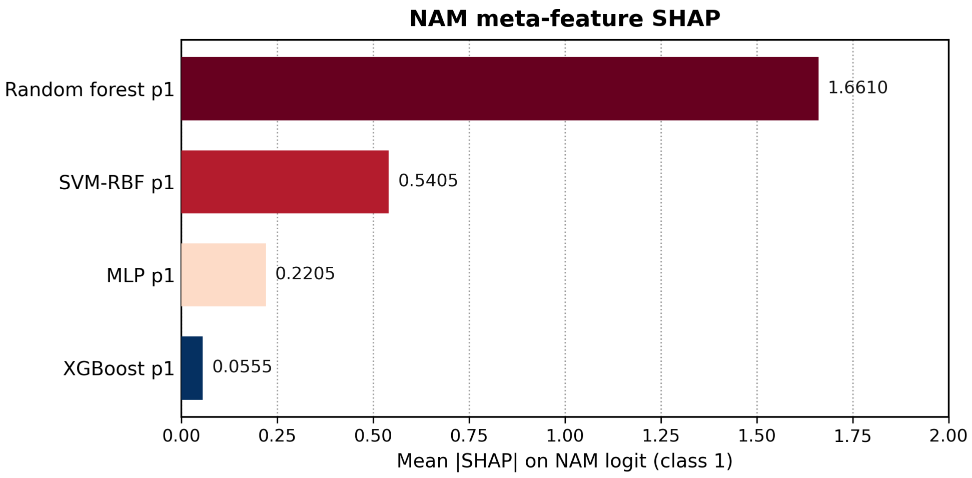

Figure S11: NAM meta-feature SHAP for the separately trained Dataset A protein-only stacking model. Random Forest has the largest mean absolute contribution and was therefore selected for the corresponding feature-level SHAP analysis.

Alt text: Dataset A protein-only NAM meta-feature SHAP ranks the four base learners, with Random Forest highest.

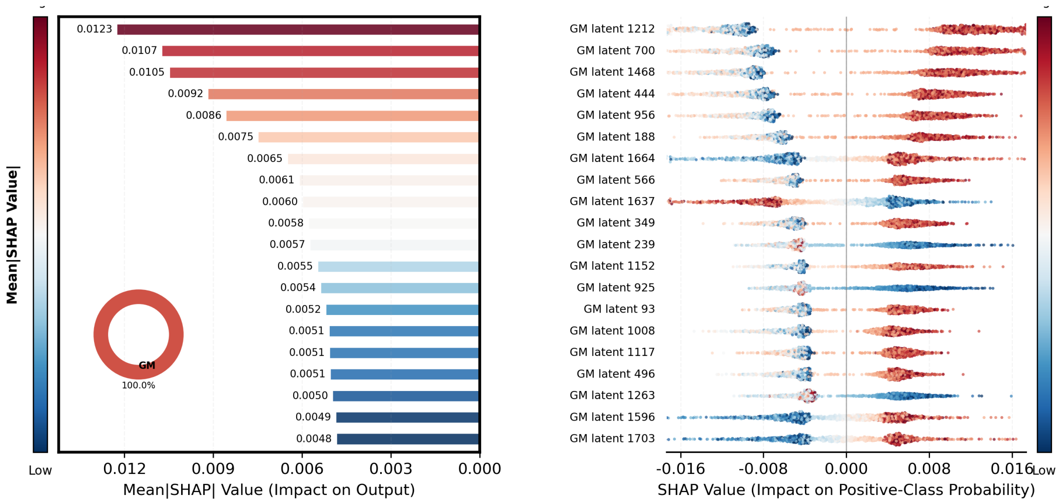

Figure S12: SHAP analysis of the separately trained Dataset A protein-only Random Forest model selected by the preceding NAM-level analysis. Learned Graph-Mamba coordinates are labeled “GM latent” and are not assigned direct residue-level or mechanistic interpretations.

Alt text: Dataset A protein-only Random Forest SHAP plot ranking Graph-Mamba latent dimensions and showing feature-level effects.

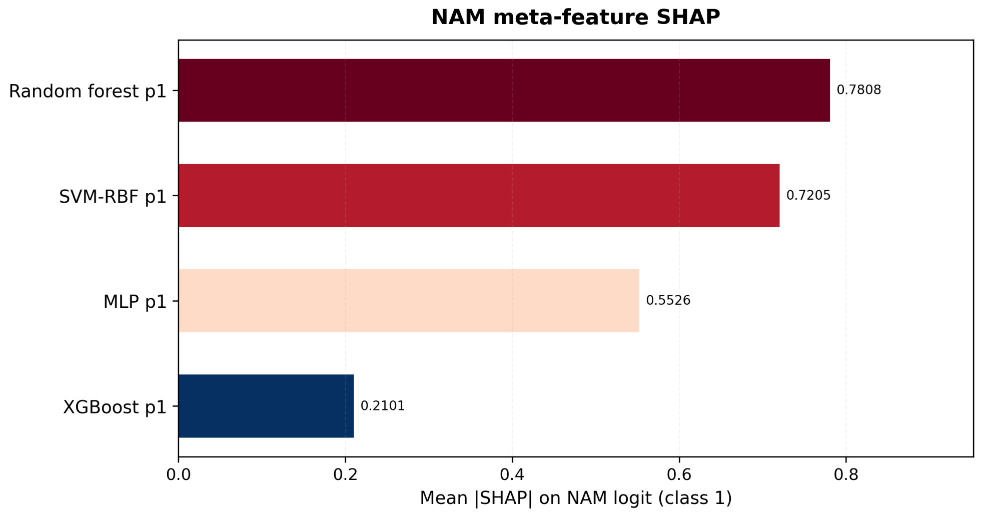

Figure S13: NAM meta-feature SHAP for the Dataset B fused stacking model. Random Forest has the largest mean absolute contribution to the NAM logit and was therefore selected for the primary feature-level analysis.

Alt text: Dataset B fused NAM meta-feature SHAP ranks the four base-learner probabilities, with Random Forest highest.

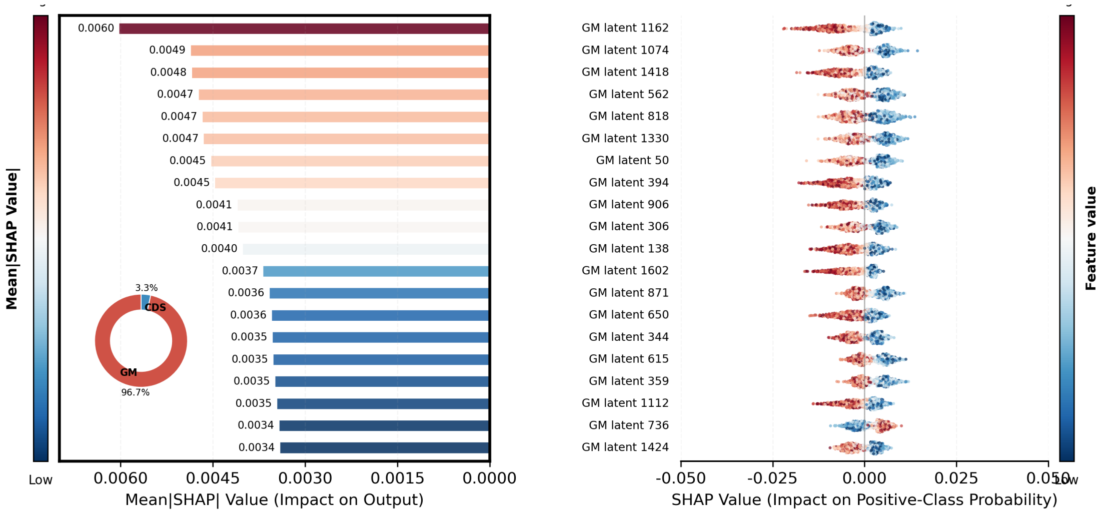

Figure S14: Primary feature-level SHAP analysis for the Dataset B fused model using the Random Forest learner selected by the preceding NAM-level analysis. The global modality donut reports CDS 3.3% and GM 96.7%.

Alt text: Dataset B fused Random Forest SHAP plot dominated by GM latent dimensions; the donut reports 3.3% CDS and 96.7% GM.

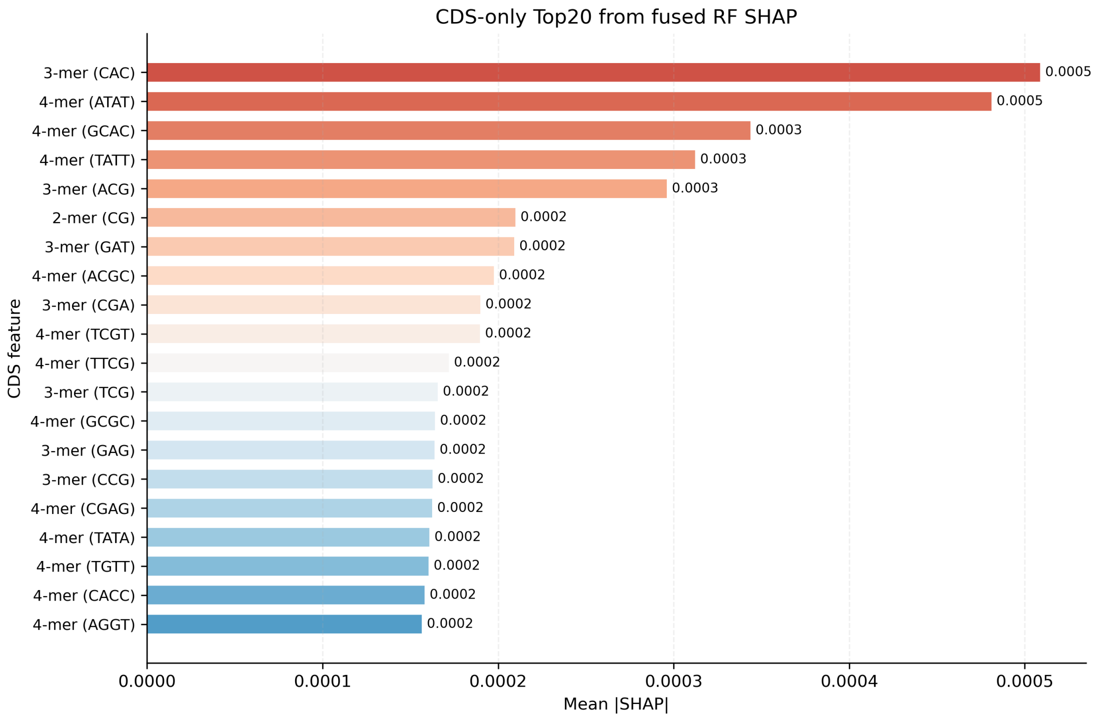

Figure S15: CDS-restricted SHAP ranking for the selected Dataset B Random Forest fused model, highlighting interpretable CDS variables including 3-mer (CAC), 4-mer (ATAT), 4-mer (GCAC), 4-mer (TATT), and 3-mer (ACG).

Alt text: Dataset B fused Random Forest SHAP ranking restricted to CDS-derived inputs, highlighting explicit 3-mer and 4-mer variables.

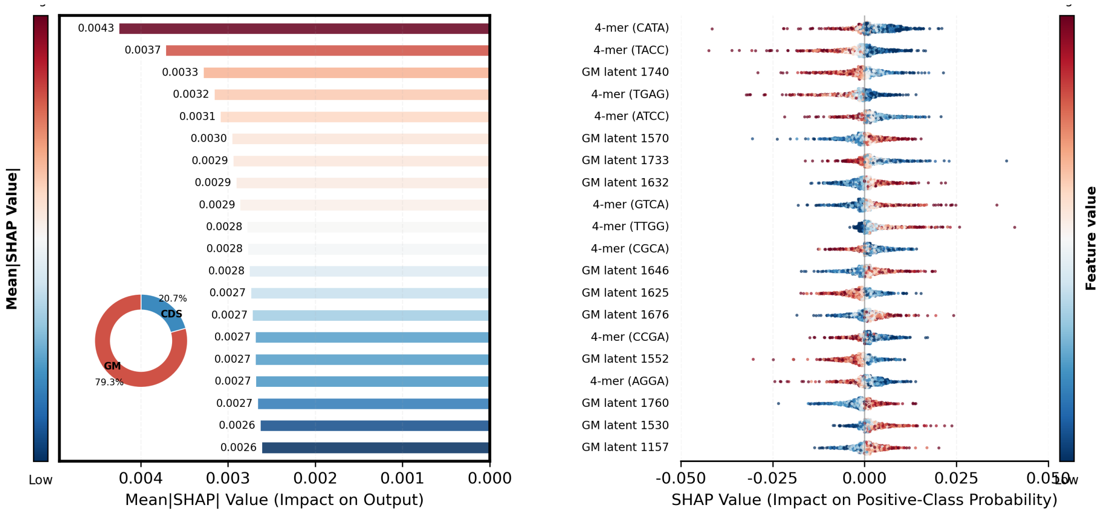

Figure S16: Fused-model SHAP sensitivity analysis for the Dataset B MLP learner. The top-ranked features include CDS-derived k-mers and GM latent dimensions; the global modality donut reports CDS 20.7% and GM 79.3%.

Alt text: Dataset B fused MLP SHAP sensitivity plot showing CDS k-mers and GM latent dimensions; the donut reports 20.7% CDS and 79.3% GM.

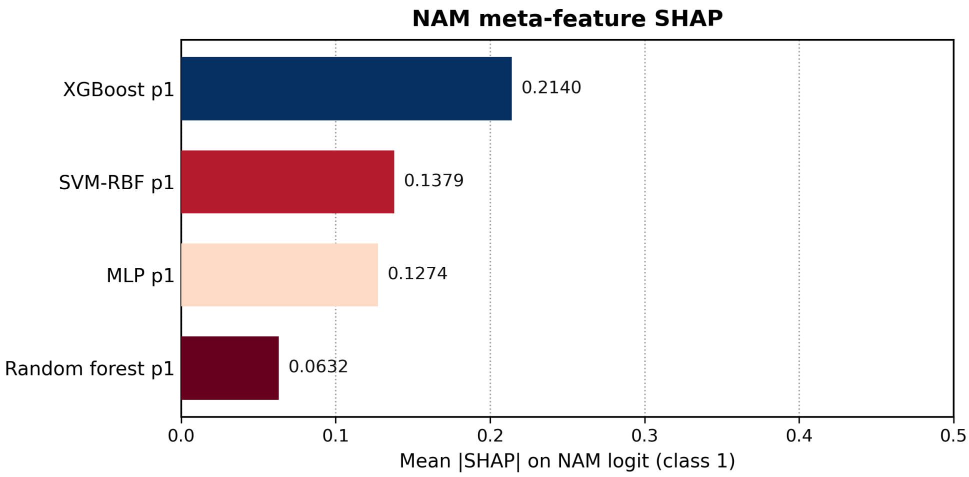

Figure S17: NAM meta-feature SHAP for the separately trained Dataset B CDS-only stacking model. XGBoost has the largest mean absolute contribution and was therefore selected for the corresponding feature-level SHAP analysis.

Alt text: Dataset B CDS-only NAM meta-feature SHAP ranks the four base learners, with XGBoost highest.

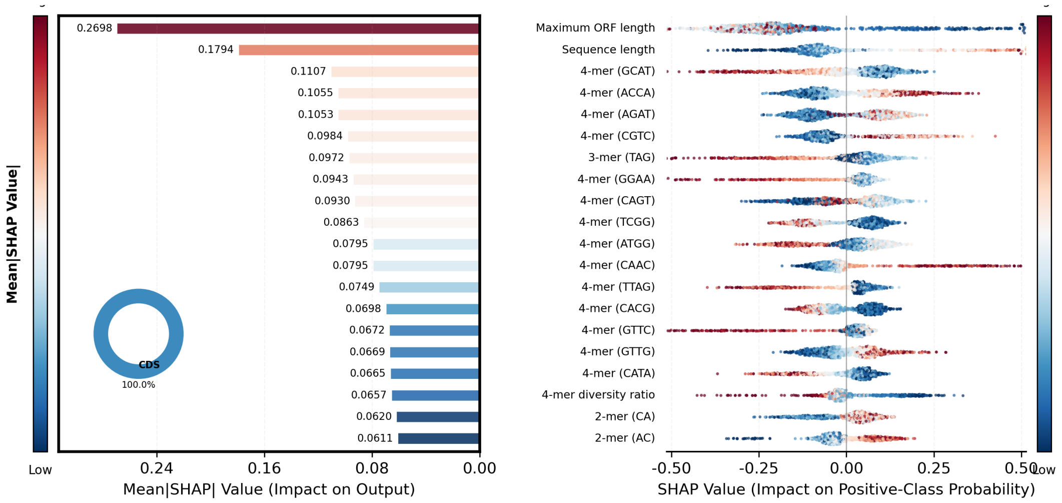

Figure S18: SHAP analysis of the separately trained Dataset B CDS-only XGBoost model selected by the preceding NAM-level analysis. CDS variables are labeled with their original sequence descriptors.

Alt text: Dataset B CDS-only XGBoost SHAP plot ranking explicit nucleotide-derived variables and showing feature-level effects.

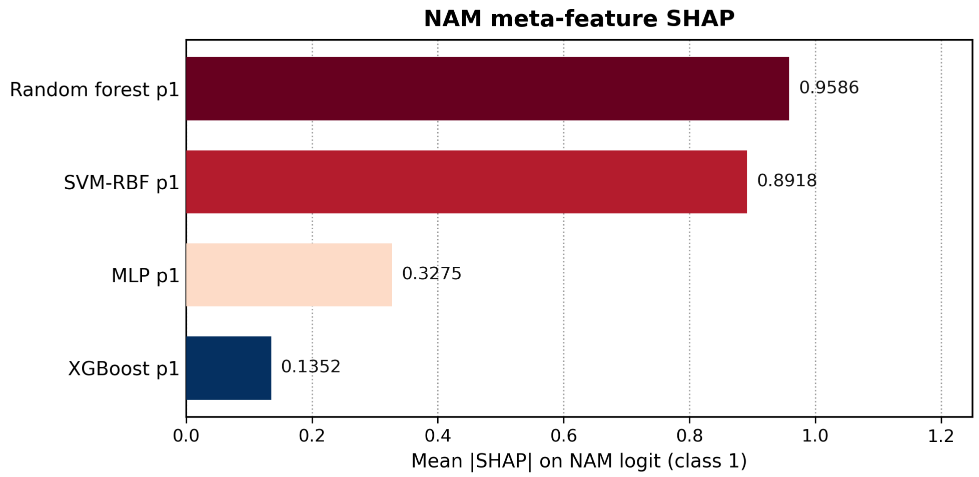

Figure S19: NAM meta-feature SHAP for the separately trained Dataset B protein-only stacking model. Random Forest has the largest mean absolute contribution and was therefore selected for the corresponding feature-level SHAP analysis.

Alt text: Dataset B protein-only NAM meta-feature SHAP ranks the four base learners, with Random Forest highest.

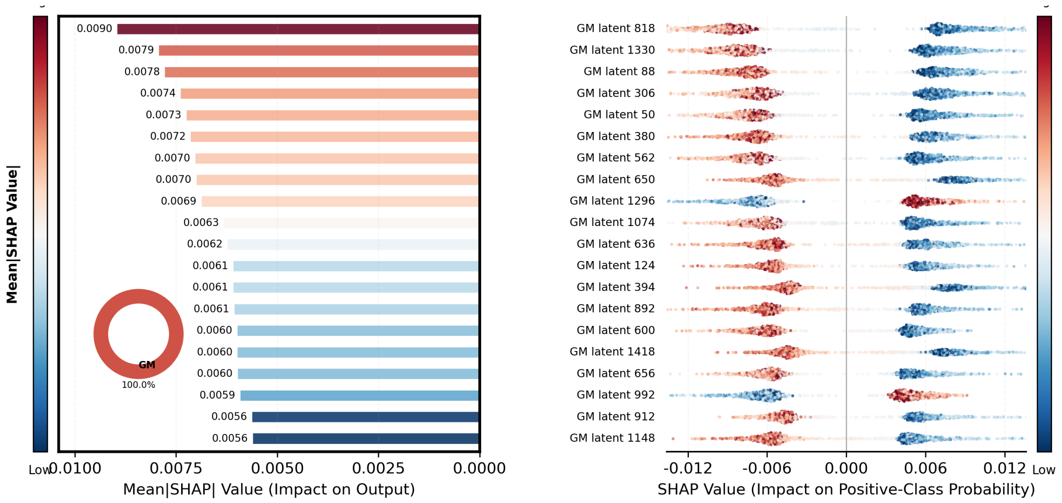

Figure S20: SHAP analysis of the separately trained Dataset B protein-only Random Forest model selected by the preceding NAM-level analysis. Learned Graph-Mamba coordinates are labeled “GM latent” and are not assigned direct residue-level or mechanistic interpretations.

Alt text: Dataset B protein-only Random Forest SHAP plot ranking Graph-Mamba latent dimensions and showing feature-level effects.

Supplementary Data

The following machine-readable files accompany the Supplementary Information.

Supplementary Data S1. Complete family composition by dataset, split, and VF/non-VF label for Datasets A and B.

Supplementary Data S2. Complete genus composition by dataset, split, and VF/non-VF label for Datasets A and B.

Supplementary Data S3. Complete species composition by dataset, split, and VF/non-VF label for Datasets A and B.

Supplementary Data S4. Complete within-family, within-genus, and within-species independent-test performance metrics for all eligible taxa in Datasets A and B.
